# Development of Air Quality Boxes Based on Low-Cost Sensor Technology for Ambient Air Quality Monitoring

**DOI:** 10.3390/s22103830

**Published:** 2022-05-18

**Authors:** Paul Gäbel, Christian Koller, Elke Hertig

**Affiliations:** 1 Regional Climate Change and Health, Faculty of Medicine, University of Augsburg, Universitätsstraße 2, 86159 Augsburg, Germany; paul.gaebel@med.uni-augsburg.de; 2 Faculty of Design, Hochschule München, Lothstraße 34, 80335 Munich, Germany; ckoller@hm.edu

**Keywords:** electrochemical sensors, metal oxide semiconductor sensors, particulate matter sensors, urban air quality, smart environment monitoring (SEM)

## Abstract

Analyses of the relationships between climate, air substances and health usually concentrate on urban environments because of increased urban temperatures, high levels of air pollution and the exposure of a large number of people compared to rural environments. Ongoing urbanization, demographic ageing and climate change lead to an increased vulnerability with respect to climate-related extremes and air pollution. However, systematic analyses of the specific local-scale characteristics of health-relevant atmospheric conditions and compositions in urban environments are still scarce because of the lack of high-resolution monitoring networks. In recent years, low-cost sensors (LCS) became available, which potentially provide the opportunity to monitor atmospheric conditions with a high spatial resolution and which allow monitoring directly at vulnerable people. In this study, we present the atmospheric exposure low-cost monitoring (AELCM) system for several air substances like ozone, nitrogen dioxide, carbon monoxide and particulate matter, as well as meteorological variables developed by our research group. The measurement equipment is calibrated using multiple linear regression and extensively tested based on a field evaluation approach at an urban background site using the high-quality measurement unit, the atmospheric exposure monitoring station (AEMS) for meteorology and air substances, of our research group. The field evaluation took place over a time span of 4 to 8 months. The electrochemical ozone sensors (SPEC DGS-O3: R^2^: 0.71–0.95, RMSE: 3.31–7.79 ppb) and particulate matter sensors (SPS30 PM1/PM2.5: R^2^: 0.96–0.97/0.90–0.94, RMSE: 0.77–1.07 µg/m^3^/1.27–1.96 µg/m^3^) showed the best performances at the urban background site, while the other sensors underperformed tremendously (SPEC DGS-NO2, SPEC DGS-CO, MQ131, MiCS-2714 and MiCS-4514). The results of our study show that meaningful local-scale measurements are possible with the former sensors deployed in an AELCM unit.

## 1. Introduction

Worldwide, one of the greatest and most challenging problems is the degradation of air quality, especially when caused by human activities. In the year 2019, according to the World Health Organization (WHO), 99% of the Earth’s population was living in regions where the air quality guideline levels given by the WHO were not achieved [1].

Recently, the WHO global air quality guidelines were updated based on the latest systematic reviews of exposure-response studies [2]. For the classical air pollutants, which include particulate matter (PM), ozone (O_3_), nitrogen dioxide (NO_2_), carbon monoxide (CO) and sulfur dioxide (SO_2_), an improved assessment of their adverse effects on health in low-, mid- and high-income countries took place in the last 15 years, achieved, for instance, through the progress in measurements, modelling, data availability and exposure analysis [2]. Olschewski et al. have shown that high-mortality events of patients with cancer are strongly linked with above-average levels of NO_2_ and PM2.5 during unfavorable weather conditions in late winter until spring, which leads to the accumulation of polluted air [3]. Generally, weather conditions and the atmospheric state influence the transport, mixing ratio, transformation and deposition of air substances; hence they are important factors defining the air quality level [4,5,6]. Many studies show that short- as well as long-term exposures to O_3_ are associated with a higher morbidity and mortality [7,8,9]. Moreover, major evidence exists that cardiovascular mortality and morbidity is related to PM exposure [10,11,12]. CO, a highly poisonous gas [13], is another air pollutant detrimental to human health, being associated with acute respiratory and cardiovascular diseases, for example [14,15]. The combination of the high impact of ambient air pollution on human health with a still not satisfactory spatial resolution of air quality monitoring worldwide points at the need for an increase in measurement resolution of health-deteriorating air pollutants. The lack of high-resolution air pollutant monitoring is due to the high initial and maintenance costs of regular devices for monitoring air pollution [16,17,18].

Therefore, it is reasonable to aim at developing custom multipollutant air-quality monitoring systems to characterize the variation of those classic pollutants in a high spatial resolution and which are also far less cost-intensive compared to official measurement devices. In the last decade, an increasing number of companies started to produce low-cost sensors (LCS) or even whole sensor systems (SSys), which include supporting components (e.g., enclosure, power supply, hardware and software for data treatment). This also sparked the endeavors of researchers to build low-cost air quality monitoring networks as a complementary source of information [16]. While these manufactured sensor systems were evaluated, for instance, through collocation experiments [18], researchers also evaluated the sole sensors or sensor systems embedded in their own custom-built measurement systems [19,20,21,22]. In this work, we do the latter and introduce the atmospheric exposure low-cost monitoring (AELCM) device, which can deliver high spatial and temporal resolution data of air pollutant concentrations and climate parameters for Internet of Things (IoT) applications using an Arduino and Pycom microcontroller board.

Generally, lower-cost monitoring devices for gaseous pollutants in ambient air use electrochemical sensors (EC) or metal oxide sensors (MOS), while optical particle counters (OPC) are used the most for the detection of PM [23]. All these sensor types are incorporated in the first version of the AELCM, which also includes a low-cost sensor for the measurement of temperature and humidity inside the box. The sensor output can be highly dependent on the variation of these meteorological variables [24,25,26]. The incorporated sensors in the AELCM box are supposed to measure the classic pollutants PM2.5, NO_2_, O_3_ and CO. While low-cost sensors offer many potential advantages, there are also major disadvantages. Their overall short lifespan, lack of long-term stability, signs of inter-sensor unit variability, cross-sensitivity and the need to calibrate them using reference instruments because of the lack of sufficient conversion formulas for individually purchased sensors are detrimental to their overall use in a systematic and beneficial way [16].

In addition to a technical presentation of the AELCM, we also want to address the performance of the used sensors (some of them are rarely evaluated, such as the digital gas sensors manufactured by SPEC Sensors) in the context of some of the issues these sensors entail. For this purpose, we are using the reference measurements of the atmospheric exposure monitoring station (AEMS) for air substances and meteorological variables, while our AELCM boxes are mounted next to the station.

## 2. Materials and Methods

### 2.1. AELCM Design and Collocation Experiment

The first version of the atmospheric exposure low-cost monitoring box uses an Arduino Mega 2560 Rev3 board for the collection of sensor measurements. Moreover, the Arduino device is used for the control of the other embedded hardware in the AELCM box. The board uses an ATmega 2560, which is a power-efficient and performant 8-bit microcontroller [27].

The Arduino Mega 2560 provides a wide variety of features, while the most important features for our measurement system were the 16 MHz crystal oscillator, an available USB connection, the high number of digital input/output pins and analogue input pins, as well as the amount of memory. For communication with external devices, such as a secure digital (SD) card module (which enables storage of measurement data on a memory card) or a real-time clock (RTC), the board provides SPI, four UARTs (hardware serial ports) and I2C. The synchronous serial data protocol called the serial peripheral interface (SPI) is used to communicate with the SD card module and to control it. The universal asynchronous receiver-transmitter (UART) protocol and the serial communication bus I2C (inter-integrated circuit) are two additional communication protocols being used. The connection between the board and its external devices was mostly realized through different self-designed printed circuit boards (PCBs). These custom-built PCBs included a so-called bus board, a sensor board and a communication board. The PCBs have individual slots for different hardware (for instance, sensors, SD card module, data modem and optional GPS module).

Data transmissions and regular syncs of the external Adafruit DS3231 RTC are realized through a GPy board by Pycom based on the Espressif ESP32 SoC (System on Chip) [28]. The external real-time clock is used to guarantee that the system time of an AELCM unit is still precise, even if there is poor network connectivity. Next to location tracking, time synchronizations in countries with a lack of IoT infrastructure could be realized through the optional GPS module (Adafruit Mini GPS PA1010D). The GPy is a triple-bearer, offering connectivity in form of WiFi, Bluetooth and cellular LTE-CAT-M1/NB1. We are using long-term evolution for machines (LTE-M) to enable the AELCM box to gain a connection to the internet, which requires an IoT Nano–SIM. We are employing an IoT SIM card by the carrier 1NCE. Likewise, for transmitting data successfully we needed a communication device that supports modern security standards, such as TLS version 1.2 (Transport Layer Security). In addition, a minimal peak current consumption during transmissions was needed. Both requirements are features of the GPy. Two uninterruptable power supplies (Adafruit PowerBoost 1000C) provide current of up to 1 ampere at a voltage between 5 and 5.2 volts (V) for the GPy and the rest of the measurement system via the mains or batteries separately [29]. The separation exists to avoid a possible instability of an AELCM unit that is due to voltage drops caused by potential high current consumption greater than 1 ampere during data transmissions. Moreover, it provides more flexibility with respect to the operation of metal oxide sensors, which consume vastly more current by design because they use heaters in contrast to electrochemical gas sensors. The integrated LCD is used for more comfortable deployment in the field, confirming if the boot up of an AELCM unit is successful or if hardware components are failing.

**Figure 1:**
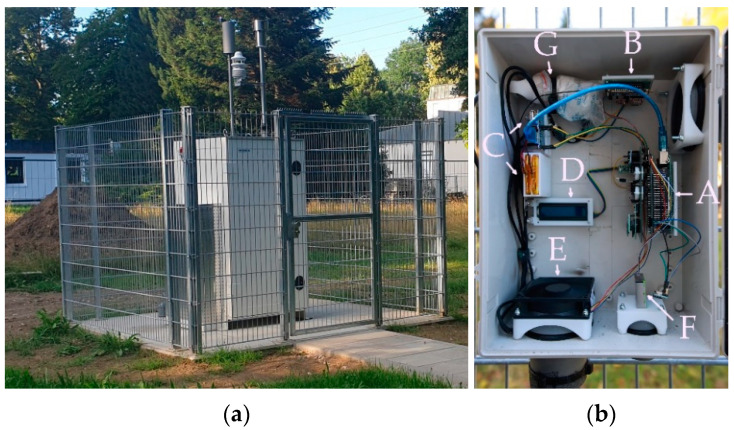
Photographs of the AEMS and an AELCM unit, which is mounted on the fence next to the AEMS: (**a**) the stationary air and climate measurement station of the Chair for Regional Climate Change and Health, Faculty of Medicine, University of Augsburg; and (**b**) the housing of an AELCM unit consists of a weather-proof NEMA (National Electrical Manufacturers Association) enclosure (ABS Plastic Case, PN: NBB-22251; L × W × H: 37 cm × 27 cm × 15 cm). The unit includes a sensor board (equipped with gas sensors and a meteorological sensor) connected to a bus board, which in turn is connected to an Arduino Mega 2560 Rev3 and an external RTC (A). The communication board is equipped with an antenna-equipped Pycom GPy and an SD card module (B). An uninterruptable power supply for the communication device (GPy) and a second one for the measurement system and its external devices (LCD, fan and SD card module) are each connected to a LiPo battery (C). A liquid crystal display (LCD) shows the status and functionality of the AELCM unit (D). The DC PWM fan inside the enclosure (ODROID-H2 DC Fan; L × H × D: 92 mm × 92 mm × 25 mm) makes use of the custom air inlet (top right) and custom air outlet (bottom left) for the exchange of air (E). The SPS30 located at the bottom of the box directly coupled to the ambient air through a short plastic pipe (F). Both uninterruptable power supplies are connected to a two-port USB charger plugged into a regular power socket (G).

The housing, shown in Figure 1b, is a modified ABS plastic box, which is actively ventilated through a fan to probe the ambient air. The fan functions as an exhaust fan, removing air from inside the box. The resulting air flow direction is toward the bottom left of the box. The fan guarantees a constant air flow over the gas sensors and the meteorological sensor, which are described in Table 1.

The revolutions per minute (RPM) of the fan blades are 2000 RPM, causing a flow velocity between 0.8 and 0.9 m/s at the end of the bottom exhaust pipe of an AELCM box [39]. 3D printing was an essential tool for customizing the box and securing the used hardware.

The gas sensors in Table 1 can be separated into two categories: electrochemical gas sensors (SPEC DGS) and metal oxide gas sensors (MiCS-2714, MiCS-4514, MQ131). Electrochemical gas sensors generate a measurable current while in contact with their target gas [40]. The amount of measured current depends on the concentration of the target gas. This relationship is utilized to estimate gas concentrations. Metal oxide sensors on the other hand feature a semiconductor layer. The conductivity of this layer changes with the fractions of the gases in the air to which a metal oxide type gas sensor is sensitive [16]. We can measure this change and translate it into estimations of gas concentrations of a specific gas. For the measurement of PM, an optical particle counter (OPC) was used, called SPS30. Through the interaction of laser beams and particles, resulting in scattered light inside a measurement cell collected by a photodetector, the mass concentration of suspended matter with diameters smaller than or equal to specific aerodynamic diameters (PM2.5 and PM1) can be estimated [38].

The measurement of the meteorological parameters of relative humidity and temperature are relevant for analyzing LCS output. The neglect of meteorological conditions can lead to a sensor output and in consequence to model-estimated concentration levels, which can be questionable as mentioned in the introduction.

In our configuration, the measurements of these two variables taken by a BME280 are an integral part of the model-based output adjustments of the sensors embedded in an AELCM unit. The possible influence of these parameters on the sensor outputs given by electrochemical- and metal oxide-type sensors are also evident in the official documents of manufacturers who produce these types of sensors [31,40].

The Arduino board, a device with a 5 V logic level, was interfaced with the digital gas sensors (DGS) by SPEC sensors using UART hardware serial ports and logic-level converters, given that these sensors are devices operating on 3.3 V. These units give gas concentrations in parts per billion (ppb) and were rarely analyzed in regards of their performance in reports or other scientific literature in the past compared to the electrochemical sensors of the manufacturer Alphasense (e.g., B4-series and A4-series), which are up-to-date, common sights in experiments or low-cost sensor systems for gaseous air pollutants [22,23,41]. Therefore, we have decided to evaluate the performance of these sensors as well. The communication between the SPS30, BME280, Adafruit ADS1115, Adafruit DS3231 and Arduino board was realized by I2C. The analogue sensors MQ131, MiCS-2714 and MiCS-4514 were connected to the Adafruit ADS1115 analog-to-digital converter (ADC) to read their outputs.

AELCM units were collocated with the air and climate measurement station of the Chair of Regional Climate Change and Health next to the University Hospital Augsburg in an urban background setting shown in Figure 1a named AEMS (location: 48° 23.04′ N, 10° 50.53′ E; Germany). The measurement station and its instruments are provided by the company Horiba (APOA-370, principle: non-dispersive ultra-violet-absorption method (NDUV), APNA-370, principle: chemiluminescence method (CLD), APMA-370, principle: non-dispersive infrared (NDIR) absorption method, APDA-372, principle: optical light-scattering). The AEMS provides measurements of the air constituents O_3_, nitrogen oxides (NO, NO_2_ and NO_x_), CO and fine dust (PM1, PM2.5, PM4 and PM10). Additionally, the AEMS is equipped with a Lufft WS600-UMB, which measures the meteorological variables air temperature, relative humidity, precipitation intensity, precipitation type, precipitation amount, air pressure, wind direction and wind speed.

An internal datalogger called the µIO-Expander averages the gas concentration measurements to 3 min means, while the concentrations of PM fractions are rolling means, averaged over 15 min time intervals by the datalogger. Measurements of an AELCM unit take place every 10 s and get saved on an SD card inside an SD card module.

The Arduino board averages the collected data to 3 min means and sends them to the GPy via a software serial port established on the Arduino board. This step was necessary because all hardware serial ports (UART) are occupied by digital gas sensors. The GPy sends the averaged data in real time to our database, being embedded in the server infrastructure of the University of Augsburg.

### 2.2. Data and Data Treatment

We deployed three AELCM units with slightly different sensor configurations and different deployment dates, which are summarized in Table 2. The general analysis period ended on 24 October 2021. The longest time series is available for the unit AELCM003, which was deployed in late February 2021. The units AELCM004 and AELCM005 were deployed in June 2021. The different deployment dates are a result of the development of a custom surface-mounted device (SMD) socket for the MiCS-2714 and MiCS-4514, which was not completed by the end of February 2021 (Figures S1.1 and S1.2). A custom SMD socket was developed to avoid manually soldering the MiCS devices to the sensor board, possibly damaging them in the process.

Eventually, we decided to deploy the first AELCM box called AELCM003 without MiCS sensors. Originally, the box AELCM004 was deployed at the same date as the unit AELCM005, but we had to readjust the SMD sockets for the MiCS sensors multiple times for this box, so that the official measurement start was 21st of June for the AELCM004 unit.

The issues with the current MiCS socket made us redesign it. The latest MiCS socket board could not be implemented in the first version of the AELCM units anymore because it was not compatible with the current sensor boards in the already deployed units (Figures S1.3 and S1.4). We also decided not to employ any SPEC DGS-CO devices in later units anymore after looking into its initial performance in the field experiment. The reasoning behind this decision is given in Section 3.

Overall, saving the measurement data with the microSD breakout by the manufacturer SparkFun on a SD memory card worked well. We could retrieve all the measurement data for the AELCM units 003 and 004. Unfortunately, the SD memory card of AELCM005 stopped functioning, so the raw data for this measurement system were not available (n. A.). Nevertheless, we could still use the 3 min averages saved to our database.

During the field experiment, the GPy boards were able to send most of the averaged measurement data to our database. Approximately 95% or more of the averaged data were successfully sent. The missing averages in the database can be explained with ongoing network issues on the carrier side or with a rare problem, where the GPy is losing its internet connection and cannot recover solely with a board reset. Only a manual power cycle resolved this issue. We recommend integrating a metal–oxide–semiconductor field-effect transistor (MOSFET) controlled by a microcontroller or microcontroller board, such as in our case an Arduino board, to power-cycle the GPy automatically in those cases.

While called SPEC DGS-O3, this EC sensor has a 1:1 sensitivity to NO_2_; hence, it must be deployed with a NO_2_ sensor to estimate the concentration of O_3_ correctly [34]. Thus, the AEMS measurements for NO_2_ were subtracted from the measurements of the DGS-O3 units. Most of the data sheets of the different employed gas sensors recommend a warm-up time before actually using the sensor output for estimates. The digital gas sensors by SPEC sensors show a startup output profile after getting powered. The length of the output stabilization process may depend on the length of time the sensor was unpowered or the sensor type [34,35,36]. The observed stabilization periods of the digital outputs were different between the DGS used in the field experiment, but in general were shorter than a day. On that account, the first 24 h of measurements of the SPEC DGS devices were not included in this analysis. Another important detail is that the digital gas sensors and even the AEMS gas measurement devices show a slight degree of noise in their measurements. This is a normal feature inherent to their technical design and becomes most evident in negative gas-concentration measurements (DGS, AEMS). Consequently, negative hourly means of gas concentrations were flagged and set to zero ppb.

The noise levels for the AEMS gas measurement devices have been kept between ±0.5 ppb (APOA, APNA) and ±10 ppb (APMA) through recalibrations. Subtracting the AEMS NO_2_ levels from the output of the DGS-O3 could also result in negative ppb concentrations, showing that there are limitations in sensing lower levels of O_3_ and/or NO_2_ with the DGS-O3. A warm-up phase also exists for the MQ131 according to its data sheet. Because we do not know how long a sensor was not operated after being manufactured and stored at a sensor distributor’s warehouse, we decided to exclude a week of sensor data from the MQ131 before evaluating its actual output. This amount of removed data is related to a storage time of more than six months for the MQ131 [31]. The data sheets for the MiCS sensors do not provide any information about a needed warm-up duration. Consequently, we also removed the first week of measurements of the MiCS sensors. Our decision is based on the data sheet of the MQ131 (another metal oxide sensor), which was described earlier. All low-cost gas sensor data were averaged to hourly means, while the sensor data of the low-cost PM sensor SPS30 were averaged to 15 min means. Since the SD memory card data were not available for the unit AELCM005, we used the transmitted 3 min averages for this unit. For this measurement system, hourly means and 15 min means were only calculated, when all data were available for the averaging process, otherwise an hourly mean or 15 min mean was flagged as a missing value. Furthermore, we limited the evaluation of the SPS30 to its output for the fractions PM1 and PM2.5 because of its technical limitation to provide proper estimates for the fractions PM4 and PM10 [38]. We also removed the first 24 h of measurements for the SPS30 after observing on multiple occasions that there is an initial stabilization period (the PM2.5, PM4 and PM10 measurements become identical after some time) after powering this device.

The 3 min averages for the gaseous air pollutants given by the reference measurement station AEMS were averaged to hourly means. Because the concentrations of the PM fractions are provided as rolling averages by the AEMS over a time span of 15 min, we extracted the data at the minutes 0, 15, 30 and 45 for every hour (e.g., 14:00, 14:15, 14:30 and 14:45 CET) and used these as a reference basis for the evaluation of the SPS30. The system time of the AEMS is CET, while the system time of the AELCM units is UTC. Subsequently, we adjusted the time stamps of the AELCM measurement data to CET. Portions of the reference data had to be excluded or were not available. The reason is, on the one hand, regular maintenance work involving the power grid of the University Hospital, where no power is available to operate the measurement station. On the other hand, the AEMS must be regularly maintained and checked (e.g., feeding of reference gas or reference dust, recalibration of measurement devices and filter exchange) to guarantee a reliable operation and high-quality data output.

### 2.3. Methods

#### 2.3.1. Calibration

We built multiple linear regression models (MLR) for the LCS to calibrate the hourly means of the gaseous sensor data and 15 min means of PM sensor data. We did not expect that adding every possible predictor in our MLR models would result in a significant improvement of the model, so we selected a reasonable set of predictors using the following steps: (1) using a Spearman rank correlation to obtain a first general impression about the strength of the relationship between low-cost sensor output and reference measurements; (2) building MLR models (calibration functions) for each sensor based on their data output considering environmental influences on sensor output and reference measurements (AEMS); and (3) predictor selection for the final regression models for every individual LCS deployed based on the found models in step (2). The last step is realized through a stepwise regression involving a sequential replacement algorithm and an out-of-sample (OOS) approach using the RMSE as an evaluation parameter. The sequential replacement algorithm was provided by the package leaps in statistics software R [42]. The final regression models are introduced in Section 2.3.4 for every LCS. For the development of the MLR models in step (2), we start with a common set of predictors for each model, which includes the sensor data output, atmospheric humidity and air temperature. This can be regarded as the simplest model realization (baseline model, see Equation (3)). We adjust this baseline model where necessary, considering the usual MLR model assumptions, including the inspection of the residuals.

It must be mentioned that a common problem of a linear regression on atmospheric time-series data with a high temporal resolution is also apparent in this analysis. A standard assumption for building an MLR model is independent residuals, which is violated in this work. Additionally, multicollinearity (e.g., between air temperature and relative humidity) is another common issue within the subject of modelling air pollution based on LCS data. Air temperature also has a positive effect on the buildup of O_3_ near the surface. Nevertheless, we are using MLR models because MLR is still the most common basic approach for building calibration functions to process LCS data given by new measurement systems such as our AELCM unit before developing or approaching more sophisticated calibration models in future works [23,41,43]. We thoroughly evaluate every final regression model and estimate the RMSE and coefficient of determination as recommended in other literature [23].

#### 2.3.2. Evaluation Statistics

To obtain a first guess about the (monotonic) relationship between the raw *LCS* output and the reference data given by the *AEMS* and the meteorological parameters, we use the Spearman rank correlation [44]. The Spearman rank correlation is defined as:

(1) $$r_{rank}=\frac{Cov\left(R\left(LCS_{Raw}\right),R\left(AEMS_{ref}\right)\right)}{\sigma_{R\left(LCS_{Raw}\right)}\sigma_{R\left(AEMS_{ref}\right)}},$$

given by the standardized covariance of the ranked data values of the raw *LCS* measurements $\left(R\left({\mathrm{LCS}}_{\mathrm{Raw}}\right)\right)$ and ranked observations of the *AEMS* $\left(R\left({\mathrm{AEMS}}_{\mathrm{ref}}\right)\right)$. The standard deviations that belong to these ranked data values are $\sigma_{R\left({\mathrm{LCS}}_{\mathrm{Raw}}\right)}$ and $\sigma_{R\left({\mathrm{AEMS}}_{\mathrm{ref}}\right)}$, respectively.

For evaluating the calibration functions, we use two measures of the model fit: coefficient of determination (R^2^) and the root-mean-square error (*RMSE*) [44]. R^2^ can be understood as a measure of the proportion of variation of the predictand accounted for or described by a regression model. The mean squared error (*MSE*) is the arithmetic average of the squared difference between predictions and observations, reflecting the average forecast accuracy. The *MSE* is defined as:

(2) $$MSE=\frac{1}{N}\overset{N}{\underset{i=1}{\sum }}{\left(LCS_{adj_{i}}-AEMS_{ref_{i}}\right)}^{2},$$

where ${\mathrm{LCS}}_{{\mathrm{adj}}_{i}}$ and ${\mathrm{AEMS}}_{{\mathrm{ref}}_{i}}$ describe the *i*th pair of *N* pairs of model-adjusted *LCS* measurements and observations of the *AEMS*. The *MSE* is particularly sensitive to outliers caused by the squaring of errors. We use the *RMSE*, which can be expressed as the square root of the *MSE*, $\mathrm{RMSE}=\sqrt{\mathrm{MSE}}$, to describe the error between calibration (adjusted) data and reference data.

#### 2.3.3. Stepwise Regression

For a given air pollutant LCS, we are building linear regression models (MLR) as calibration functions for the LCS output. Hence, we are using the raw LCS output and the meteorological measurements of air temperature and relative humidity as predictors and the AEMS output as the predictand. We are screening the group of predictor candidates using a stepwise regression method to decide upon which and how many predictors to use. A stepwise regression is an automated filtering procedure that follows systematic rules in adding or removing variables with predictive power from a regression model according to a selection criterion [44,45]. After every regression step (final found *p* predictor model), an out-of-sample (OOS) procedure is performed.

The autocorrelation functions for the hourly and 15 min means of the AEMS data show an autocorrelation pattern for every pollutant (Figure S2.1). This indicates that the observational data at the analyzed time scales were dependent. Cerqueira et al. show that an OOS approach is an appropriate choice for model validation under this circumstance [46]. For this reason, we rely on the OOS approach to evaluate the models, considering the apparent dependence structure of the data.

Summarizing this method, which is described in detail in [46], a random point *t* in time (e.g., 10 September 2021 12:00:00 CET) of the time series *ts* is chosen to separate the training and evaluation data. The previous window with reference to *t* comprising 60% of *ts* is used for training and the following window of 10% of *ts* is used for testing. For 10 repetitions, we receive 10 randomly chosen dates *t*, which separate the training and evaluation sets. The sizes of the training and evaluation sets depend on the length of the available LCS time series and reference data (see Table 2 and Section 2.2). Finally, considering the average, minimum and maximum RMSE for the training and evaluation period for every *p* predictor model, we have chosen the final regression equation for a LCS (S3). In the case different predictive variables for a *p* predictor model were chosen between repetitions, the most chosen predictor combination defined the final *p* predictor regression model. In addition, results of a fivefold cross validation (CV) are included in the supplement of this study because of its common use in model evaluation (S4). The conclusions drawn from the CV approach are similar to the findings for the OOS approach in this study.

#### 2.3.4. Regression Models

First, we introduce the baseline linear regression model:

(3) $$LCS_{adj}=b_{0}+b_{1}LCS_{Raw}+b_{2}T+b_{3}RH,$$

where ${\mathrm{LCS}}_{\mathrm{Raw}}$ is the raw output of the low-cost pollutant sensor, while T and RH are temperature and relative humidity, respectively, given by the meteorological low-cost sensor inside the AELCM unit. ${\mathrm{LCS}}_{\mathrm{adj}}$ is the model-predicted low-cost sensor output. Transformations of predictors and/or predictands are the result of a lack of homoscedasticity and/or Gaussian distributed residuals. Based on the calibration strategy introduced in Section 2.3.1, we build the following models, where:

(4) $${\left[DGS\text{-}O3\right]}_{adj}=b_{0}+b_{1}{\left[DGS\text{-}O3\right]}_{Raw}+b_{2}\frac{{\left[DGS\text{-}O3\right]}_{Raw}^{2}-1}{2}+b_{3}T+b_{4}RH,$$

is the final calibration function for all deployed *DGS-O*3 units. ${\mathrm{DGS}\text{-}O3}_{\mathrm{Raw}}$ is corrected by ${\mathrm{AEMS}}_{\mathrm{ref}}$ for NO_2_ considering that the *DGS-O*3 has a 1:1 sensitivity to O_3_ and NO_2_ ($DGS\text{-}O3_{Ox}$), so that:

(5) $${\left[DGS\text{-}O3\right]}_{Raw}={\left[DGS\text{-}O3\right]}_{Ox}-{\left[AEMS\text{-}NO2\right]}_{ref},$$

while for the *DGS-NO*2, we used the following regression models:

(6) $$\log\left({\left[DGS\text{-}NO2\right]}_{adj}\right)=b_{0}+b_{1}{\left[DGS\text{-}NO2\right]}_{Raw}+b_{2}T+b_{3}RH,$$

(7) $${\left[DGS\text{-}NO2\right]}_{adj}=b_{0}+b_{1}{\left[DGS\text{-}NO2\right]}_{Raw}+b_{2}T+b_{3}RH,$$

where the former calibration function was used for unit AELCM003 and the latter one for the other deployed AELCM units. For the deployed *DGS-CO* device the regression model

(8) $${\left[DGS\text{-}CO\right]}_{adj}=b_{0}+b_{1}{\left[DGS\text{-}CO\right]}_{Raw}+b_{2}T+b_{3}RH,$$

was chosen. For all *MQ*131, *MiCS-*2714 and *MiCS-*4514 sensors we used the following calibration functions:

(9) $${\left[MQ131\right]}_{adj}=b_{0}+b_{1}{\left[MQ131\right]}_{Raw}+b_{2}T+b_{3}RH,$$

(10) $${\left[MiCS\text{-}2714\right]}_{adj}=b_{0}+b_{1}{\left[MiCS\text{-}2714\right]}_{Raw}+b_{2}T+b_{3}RH,$$

(11) $$\log\left({\left[MiCS\text{-}4514\right]}_{adj}\right)=b_{0}+b_{1}{\left[MiCS\text{-}4514\right]}_{Raw}+b_{2}T+b_{3}RH.$$

For adjusting the measured concentrations of a *SPS*30 for the fractions PM1 and PM2.5, we used the regression model:

(12) $$\log\left({\left[SPS30\right]}_{adj}\right)=b_{0}+b_{1}\log\left({\left[SPS30\right]}_{Raw}\right)+b_{2}T+b_{3}RH,$$

for the unit AELCM003, while using the calibration function:

(13) $$\log\left({\left[SPS30\right]}_{adj}\right)=b_{0}+b_{1}\log\left({\left[SPS30\right]}_{Raw}\right)+b_{2}RH,$$

for the other units.

## 3. Results and Discussion

The environmental conditions and pollution concentrations based on hourly (15 min) means are provided in Table 3. Erroneous measurements of the ambient air temperature and relative humidity were removed. At least 85% of the data of both variables had to be available for the calculation of the mean values.

In our work, not every LCS shows the premise of being a good-quality source of information according to the employed evaluation parameters. It was evident that some LCS could not reflect the patterns in the reference data well or at all, making them factually useless. Table 4 gives a general overview about the suitability of the deployed LCS in building a low-cost monitoring network for air constituents.

Generally, the results of our field experiment indicate that only the electrochemical sensor SPEC DGS-O3 and the PM sensor SPS30 are reasonable choices for LCS used in a low-cost atmospheric exposure monitoring network.

The other electrochemical sensors, DGS-NO2 and DGS-CO, showed no capability to measure the given concentrations at the measurement station according to the coefficient of determination and the Spearman rank correlation. The correlations were close to zero for two DGS-NO2 devices. For the longest running DGS-NO2, the Spearman rank correlation only amounted to 0.18. The low predictive power of these NO_2_ sensors is also reflected in the coefficients of determination (R^2^: 0.28–0.59). Since they are not able to sense their target gas at all or only in a very weak way, they cannot describe the variability of ambient NO_2_ to a satisfying degree. The DGS-CO even showed a negative correlation (Rs: −0.25), implying that it only produced noise. Thus, we have decided not to employ any SPEC DGS-CO devices in later AELCM units anymore after looking into their initial performance in the field experiment and testing different units, which all showed the same behavior as the SPEC DGS-CO operated in the field experiment. During a field experiment done by the Air Quality Sensor Performance Evaluation Center (AQ-SPEC), out of the DGS-CO, DGS-NO2 and DGS-O3 measurement units, only for the DGS-CO was there evidence that the LCS is able to sense ambient CO [47]. The concentration levels of ambient CO at the University Hospital Augsburg were, overall, lower in our field experiment compared to the measured concentrations in the field experiment of AQ-SPEC. This implies that the DGS-CO (at least the ones purchased from sensor distributors) is not useable for sensing lower concentrations of CO (Figure S6.2).

In contrast to the results of the AQ-SPEC field evaluation experiment, we found that most of the deployed DGS-O3 were able to detect changes in O_3_ concentration levels, albeit with differences, as can be seen in the evaluation statistics in Table 4. The raw output of the DGS-O3 deployed in AELCM005 shows the strongest relationship with the reference measurements (Rs: 0.97) and the smallest error (RMSE: 3.31 ppb), and provides the highest coefficient of determination (R^2^: 0.95). As a result, it is evident that the DGS-O3 deployed with AELCM005 shows the best level of agreement with the reference measurements, while the other DGS-O3 units show a lesser performance (RMSE: 6.74–7.79 ppb; R^2^: 0.71–0.80). Therefore, we conclude that every DGS-O3 (or any other promising LCS) must be individually screened (evaluated) and calibrated before considering them for use in any monitoring application, since even identical sensors show different characteristics in response to the same environmental conditions, such as temperature, humidity and ambient O_3_ concentrations (Figure S7.1; Table 3).

**Figure 2:**
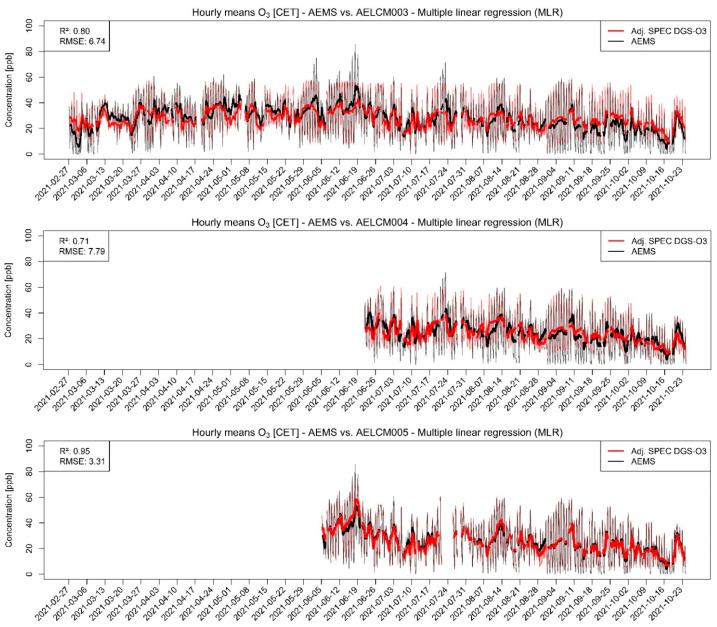
Model-adjusted hourly concentration means of different deployed SPEC DGS-O3 using multiple linear regression vs. hourly concentration means of reference measurements given by the AEMS for ambient O_3_ in ppb. The RMSE is in ppb. For smoothing the hourly concentration means, a rolling 24 h average is used: (**Top**) SPEC DGS-O3 deployed in AELCM003; (**Middle**) SPEC DGS-O3 deployed in AELCM004; and (**Bottom**) SPEC DGS-O3 deployed in AELCM005.

**Figure 3:**
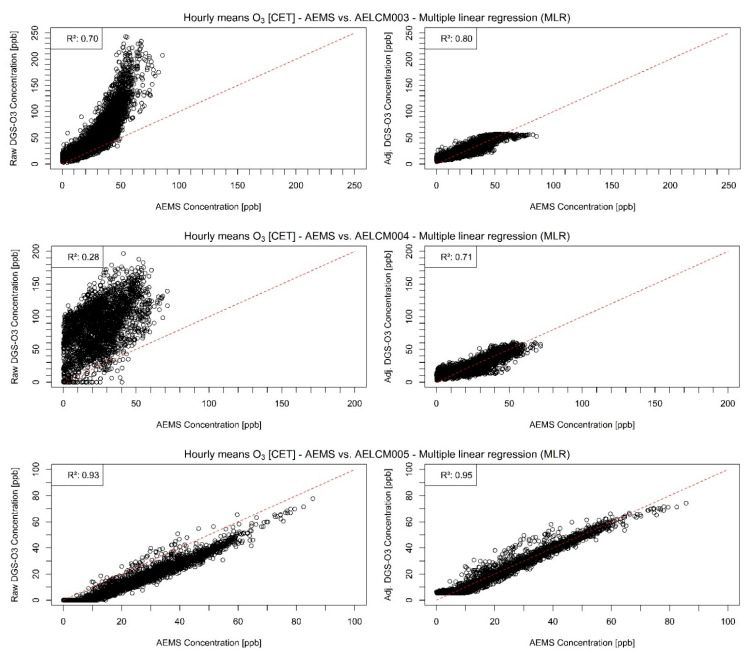
Raw (**left**) and model-adjusted (**right**) hourly concentration means of different deployed SPEC DGS-O3 vs. hourly concentration means of reference measurements given by the AEMS for ambient O_3_ in ppb: (**Top**) SPEC DGS-O3 deployed in AELCM003; (**Middle**) SPEC DGS-O3 deployed in AELCM004; and (**Bottom**) SPEC DGS-O3 deployed in AELCM005. Multiple linear regression is used for adjusting the raw LCS data.

Figure 2 and Figure 3 show the model-adjusted and the raw and model-adjusted hourly means of measured O_3_ concentrations in ppb given by SPEC DGS-O3 units deployed with the low-cost monitoring systems AELCM003, AELCM004 and AELCM005 plotted against the hourly means of the reference measurements of ambient O_3_ given by the AEMS. Additionally, the time series data in Figure 2 were smoothed using rolling 24 h averages to make deviations from the reference data more detectable. The figures for the other sensors in Supplement S6 are presented in the same manner. The AELCM boxes were deployed at different points in time because of the reasons mentioned in Section 2.2. Generally, box AELCM003 offers the longest time-series data for most of the LCS. The top panel in Figure 2 belongs to AELCM003 and indicates that the found model for the DGS-O3 describes the variability in the reference measurements reasonably well. On the other hand, quite large discrepancies between the model-adjusted LCS output and reference measurements can also be found (top panels in Figure 2 and Figure 3), resulting in a rather high error expressed through an RMSE of 6.74 ppb. The calculated average of the RMSE for the repeated holdout procedure (10 evaluation periods) is also high and amounts to 8.21 ppb (Table S3.1). Therefore, we doubt that quantitative predictions for this DGS-O3 are possible with the current model approach. Qualitative predictions in the form of an estimation of trends in the O_3_ concentration levels could still be possible with the current model approach but must be investigated in future works. The DGS-O3 deployed with AELCM004 was the worst performing sensor out of the three DGS-O3 considering the reference measurements, which is evident from the deviations between the rolling 24 h averages in the middle panel of Figure 2. The higher deviations compared to the DGS-O3 deployed in AELCM003 and AELCM005 are also shown through the RMSE statistic, which is 7.79 ppb. Considering the strength of the deviations and the only moderate relationship with the reference measurements (Rs: 0.50), a qualitative and quantitative use of the DGS-O3 deployed with AELCM004 with the current model is questionable. This is also evident from the scatterplot in the middle panel of Figure 3. Looking at the bottom panels of Figure 2 and Figure 3, a far more positive outlook for this LCS is provided through the results for the DGS-O3 of AELCM005. The DGS-O3 deployed in AELCM005 exhibits the strongest relationship (Rs: 0.97; R^2^: 0.95) and the smallest deviation from the reference measurements (RMSE: 3.31 ppb). In addition, the calculated root-mean-square errors for the predictions indicate the robustness of the final regression model (Table S3.1). The model robustness is evident by comparing the determined average, minimum and maximum RMSE for the training periods with their corresponding counterparts of the evaluation periods, which produce quite similar errors. For the selected training periods, the average RMSE is 3.09 ppb, while the individual root-mean-square errors lie between 2.49 and 3.65 ppb for the 10 training periods. For the selected evaluation periods, the average RMSE is 2.67 ppb, while the individual root-mean-square errors lie between 2.42 and 3.09 ppb for the 10 evaluation periods (Table S3.1). We would consider the DGS-O3 deployed with AELCM005 as the most promising LCS for realizing reasonable predictions of ambient O_3_ concentration using an MLR model.

Figure 3 shows clearly how differently identical LCS units can behave under the same environmental conditions and pollution concentrations. While the bottom panel shows a strong linear relationship between the raw LCS data and the reference measurements, the top panel suggests a strong nonlinear relationship with the reference. The middle panel indicates highly noisy raw LCS data of little quality. The problem of inter-sensor unit variability is thus apparent.

After discussing the electrochemical sensors, we are looking with more detail at the deployed metal oxide sensors.

While for the model-adjusted MQ131 sensor output moderate to high coefficients of determination were determined (R^2^: 0.71–0.83), the raw sensor output only shows a weak-to-moderate relationship with the reference measurements (Rs: −0.55–−0.28).

A negative relationship exists because the sensor resistance increases with higher concentrations of O_3_ [31]. The only moderate relationship for the MQ131 with its target gas reference measurements is also indicated in the stepwise selection process of predictor variables during the initial model-building process. During the model-training process, none of the MQ131 sensors could be selected as best 1-predictor model in our stepwise regression. Thus, a meteorological variable or two meteorological variables, in this case relative humidity or/and temperature, yielded a better performance than the sensor output itself. As a result, for most training periods, relative humidity was chosen as the best variable with the most predictive power in a one-predictor model. The stronger relationship between meteorological variables and ambient O_3_ is also reflected in the Spearman rank correlation between LCS measurements and reference measurements (relative humidity Rs: −0.76–−0.80; temperature Rs: 0.47–0.74; Table S5.1). Based on our analyses, we question the usefulness of the MQ131 in a low-cost monitoring network.

We draw the same conclusion for the MiCS LCS. Neither the MiCS-2714 nor the MiCS-4514 showed promising results in our field experiment considering the evaluation parameters shown in Table 4. After gaining a first impression about the usefulness of these sensors (MiCS-2714 Rs: 0.28–0.53; MiCS-4514 Rs: −0.49–0.39), their sensor output as a predictor for ambient NO_2_ and ambient CO is rather questionable. This is also reflected in the quality of the model-adjusted output of these sensors. The coefficients of determination are quite low (MiCS-2714 R^2^: 0.15–0.40; MiCS-4514 R^2^: 0.27–0.66). On the one hand, the reason could be that the overall concentration levels during the measurement campaign were too low for the sensors to sense their target gases. According to their data sheets, the MiCS-2714 has a detection range between 0.05 and 10 ppm, while the MiCS-4514 has a detection range between 1 and 1000 ppm. An ambient hourly mean of NO_2_ of 50 ppb was never reached during the measurement period of these sensors.

For the analysis periods of the deployed MiCS-4514, no hourly mean of ambient CO higher than 1 ppm was reached. Another reason could be that our former custom-built, initial prototype SMD socket for the MiCS sensors worked insufficiently, so the measurements were taken under unfavorable circumstances. The issues could be potentially resolved by our latest version, which needs to be investigated by another field experiment (Figures S1.3 and S1.4).

Considering the evaluation statistics given above for the deployed LCS, the metal oxide gas sensors performed the worst in our field experiment; therefore, we do not consider using them for future AELCM units.

Because of the poor performance of the used electrochemical and metal oxide NO_2_ LCS, we used the NO_2_ reference measurements of the AEMS to correct the raw measurements of the SPEC DGS-O3 units (see Section 2.2) before applying any calibration function.

**Figure 4:**
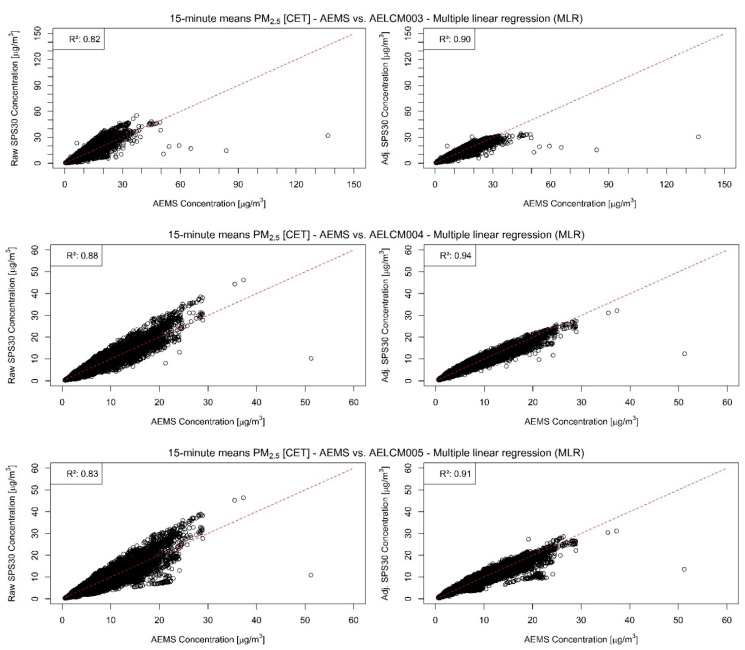
Raw (**left**) and model-adjusted (**right**) 15 min concentration means of different deployed SPS30 vs. 15 min concentration means of reference measurements given by the AEMS for PM2.5 in µg/m^3^: (**Top**) SPS30 deployed in AELCM003; (**Middle**) SPS30 deployed in AELCM004; and (**Bottom**) SPS30 deployed in AELCM005. Multiple linear regression is used for adjusting the raw LCS data.

**Figure 5:**
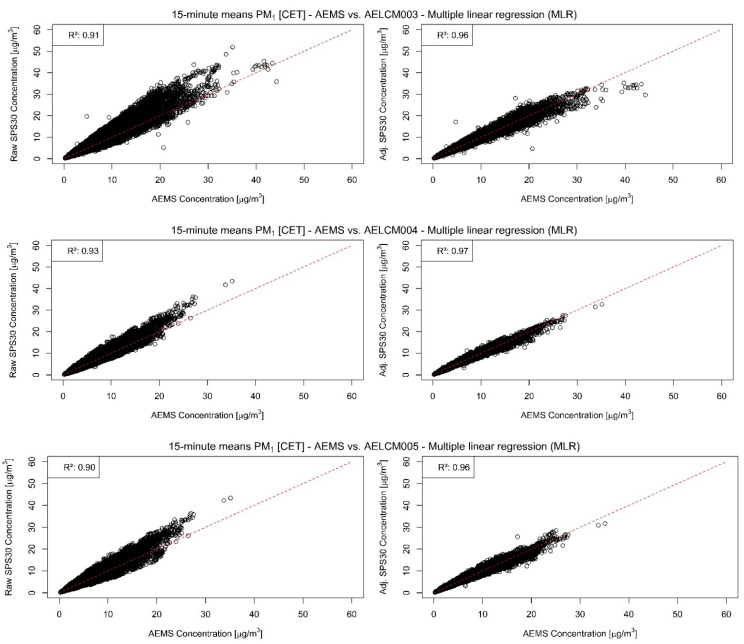
Raw (**left**) and model-adjusted (**right**) 15 min concentration means of different deployed SPS30 vs. 15 min concentration means of reference measurements given by the AEMS for PM1 in µg/m^3^: (**Top**) SPS30 deployed in AELCM003; (**Middle**) SPS30 deployed in AELCM004; and (**Bottom**) SPS30 deployed in AELCM005. Multiple linear regression is used for adjusting the raw LCS data.

**Figure 6:**
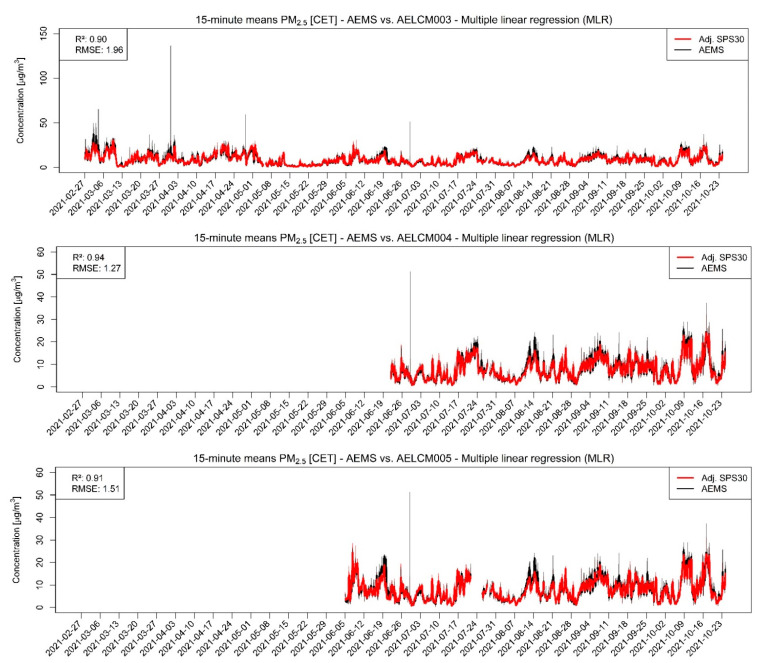
Model-adjusted 15 min concentration means of different deployed SPS30 using multiple linear regression vs. 15 min concentration means of reference measurements given by the AEMS for PM2.5 in µg/m^3^. The RMSE is in µg/m^3^. For smoothing the 15 min concentration means a rolling 24 h average is used: (**Top**) SPS30 deployed in AELCM003; (**Middle**) SPS30 deployed in AELCM004; and (**Bottom**) SPS30 deployed in AELCM005.

**Figure 7:**
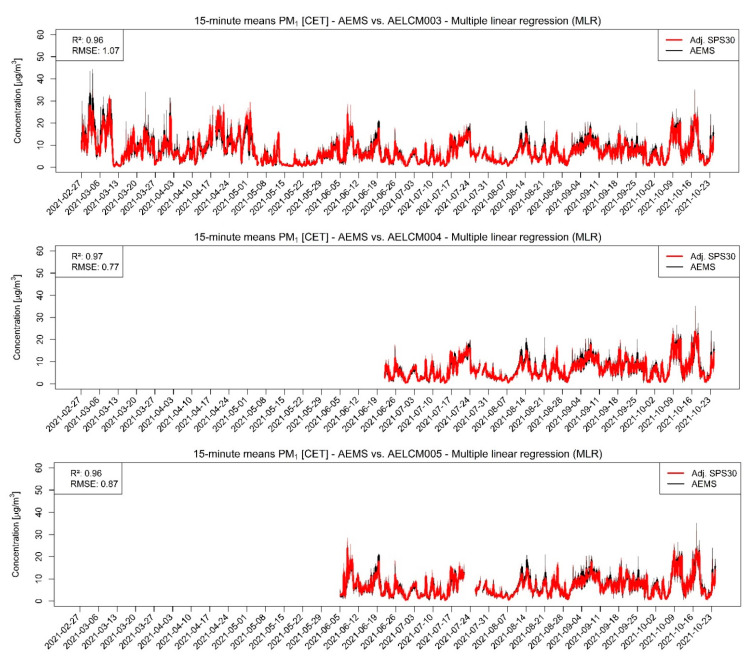
Model-adjusted 15 min concentration means of different deployed SPS30 using multiple linear regression vs. 15 min concentration means of reference measurements given by the AEMS for PM1 in µg/m^3^. The RMSE is in µg/m^3^. For smoothing the 15 min concentration means a rolling 24 h average is used: (**Top**) SPS30 deployed in AELCM003; (**Middle**) SPS30 deployed in AELCM004; and (**Bottom**) SPS30 deployed in AELCM005.

In our analyses, the PM sensor SPS30 was the best performing LCS. Each of the SPS30 devices showed for PM with aerodynamic diameters smaller than or equal to 2.5 and 1 µm strong agreements with the reference measurements, indicated through the evaluation statistics in Table 4 and in the stepwise regression results (Tables S3.7 and S3.8). The raw LCS data show a strong relationship with the reference measurements of the AEMS for both PM classes, but also an increasing spread with increasing reference measurements (Figure 4 and Figure 5). For PM2.5, the Spearman rank correlation lies between 0.94 and 0.95 and for PM1 between 0.96 and 0.97. The high level of agreement is also visible in the model-adjusted LCS output shown in Figure 6 and Figure 7, in the RMSE values and in the R^2^ values. Furthermore, the scatterplots show that the model-adjusted data align much better with the reference data. For PM2.5, the coefficients of determination are between 0.90 and 0.94 and for PM1 they are between 0.96 and 0.97. The model-adjusted LCS PM1 values describe the variability of the reference measurements slightly better than the model-adjusted LCS PM2.5 measurements.

The slightly worse agreement of model-adjusted PM2.5 values with the reference measurements is also reflected in the higher errors. These discrepancies expressed through the RMSE are between 1.27 and 1.96 µg/m^3^ but are between 0.77 and 1.07 µg/m^3^ for the model-adjusted PM1 values. The calibration functions found for the deployed SPS30 devices create good estimates overall. However, the figures and scatterplots also show that for higher PM2.5 and PM1 concentrations the error between estimate and reference can increase, resulting in underestimations of particulate matter concentrations. This behavior is less apparent for the model-adjusted PM1 concentrations.

It should be noted that we cannot explain the high peaks in some of the PM reference measurements so far. After explicitly checking the provided logs of the datalogger for these measurements producing the peaks in Figure 6, we could not find any error messages related to them, so therefore they appear to be regular measurements. Because the deployed SPS30 consistently show great agreement with the reference measurements and are deployed with the AELCM boxes just next to the AEMS, we assume that the probed air of the AEMS during these moments was highly different to the probed air by every single deployed SPS30. One of the reasons could be that suspended matter in the form of pollen could have entered the AEMS locally, which affected the measurements in these points in time, while the measurements of the deployed SPS30 did not get affected.

It should also be mentioned that for the introduced calibration functions for the raw PM1 measurements the difference between training errors and evaluation errors are generally quite low for all deployed SPS30. This indicates also that the current models seem to have a good skill for predicting PM1 concentrations, or rather for adjusting raw PM1 output of an SPS30 accordingly (Table S3.8). While the calculated RMSE values for the training and evaluation periods are higher for the model-adjusted PM2.5 measurements, we can also see similar magnitudes of errors between the training and evaluation periods (Table S3.7). Again, this indicates that the current models for adjusting raw PM2.5 concentrations given by an SPS30 are showing good skill.

The United States Environmental Protection Agency (U.S. EPA) recognizes LCS as tools that are not useable for regulatory purposes in their current state. According to the U.S. EPA, LCS technology could be potentially used as a complementary source of information, including, for example, hot-spot localization, identification of potential sites for regulatory monitoring and better understanding of local air quality [48]. For that reason, the U.S. EPA started developing performance target reports for air-quality sensors used in nonregulatory applications. The first performance-target reports for O_3_ and PM2.5 sensors were finished in 2021 [49,50]. These are based on the findings of scientific literature reviews, among other things, conducted by the U.S. EPA. We use both reports to further assess if the DGS-O3 and SPS30 could be used for nonregulatory purposes based on the calculated metrics (RMSE, R^2^) in this study for hourly (15 min) model-adjusted (corrected) data. The base testing protocol for a field test recommends an RMSE smaller than or equal to 5 ppb and an R^2^ value of at least 0.80 to consider an O3 LCS for nonregulatory supplemental and informational monitoring (NSIM) applications in ambient, outdoor and fixed-site environments [49]. For PM2.5, an RMSE smaller than or equal to 7 µg/m^3^ and an R^2^ value of at least 0.70 is needed to regard a PM LCS for NSIM purposes [50]. The DGS-O3 employed in AELCM003 and AELCM005 both fulfill the latter requirement (R^2^: 0.80, 0.95), but only for the DGS-O3 of AELCM005 is the error in an acceptable range (RMSE: 3.31 ppb) for NSIM applications. Instead of 15 min means, the recommended performance metrics and target values for PM2.5 are based on 24 h averages. Considering the individual results for the model-adjusted 15 min means of all employed SPS30 (RMSE: 1.27–1.96 µg/m^3^; R^2^: 0.90–0.94), the calculated daily averages and their corresponding performance metrics and target values fulfill the PM2.5 NSIM requirements.

For both LCS, it must be noted that not all recommended requirements for base testing were fulfilled. For instance, three O_3_ and PM LCS of the same model were not deployed at the same date (Table 2). Again, it must be said that the metrics were calculated using corrected LCS data. The U.S. EPA emphasizes in both target reports that test protocols were provided for estimating the out-of-the-box LCS performance and potential variation among identical sensors. Nevertheless, considering the recommended target values for base testing by the U.S. EPA and the calculated metrics in this study, only the SPS30 appears to be useable for NSIM applications. The DGS-O3 needs improvement to be regarded for NSIM purposes, considering its obvious strong inter-sensor unit variability.

## 4. Conclusions

In this work, besides introducing the technical aspects of our research group’s own monitoring device, the atmospheric exposure low-cost monitoring (AELCM) for meteorological variables and air constituents, we also evaluated the performance of low-cost sensors for the air pollutants O_3_, NO_2_, CO and PM with aerodynamic diameters smaller than or equal to 2.5 and 1 µm during a field experiment in an urban background area.

The overall quality and quantity of data for different air substances (PM and O_3_) and the stability of our measurement devices in the field over their individual measurement periods (4 to 8 months) show that our prototype AELCM devices are built on a solid foundation for actual individual exposure monitoring. Based on our gained experiences during the field experiment, we already included new features in our latest version of the AELCM, which will be used in future works. The new features are as follows: we eliminated the need of manual power cycling because of data modem instabilities by using a MOSFET. Additionally, it is possible to retrieve all measurement data using the file transfer protocol (FTP). This feature is especially useful for countries where the network infrastructure is not as developed for low-power data transmissions (in our instance LTE-M) as in Germany, but a degree of connectivity is needed without interrupting the measurement process of an AELCM box. Furthermore, the status of an SD memory card belonging to an AELCM unit can be checked in the database. Through this feature, we can act accordingly and replace a damaged card immediately. The already existing and latest feature set of our AELCM units and their flexibility given through a modular PCB design (easily switchable and stackable custom boards with different functions) make them promising devices for further research related to exposure monitoring.

To evaluate the sensors, we used the Spearman rank correlation for the raw measurements and a multiple linear regression approach for training and testing the sensor data. The basis of this evaluation process were the reference measurements given through high-quality measurement devices of the atmospheric exposure monitoring station (AEMS) belonging to the Chair for Regional Climate Change and Health of the University of Augsburg. Ultimately, we found that the deployed metal oxide gas sensors seem not to be useful for exposure monitoring given the circumstances in our field experiment. Either these sensors could not offer meaningful sensor output for the given pollution levels or our current sockets were too flawed in their design (not including the MQ131) to use these sensors to their full potential. The deployed electrochemical sensor for O_3_ called SPEC DGS-O3 was the only electrochemical gas sensor that showed any degree of promise, but also strong inter-sensor unit variability. It must be noted, though, that this sensor must be combined with another NO_2_ LCS to use its full potential, since its measurements get affected strongly by NO_2_. Only two of these O_3_ sensors showed promise under the aspect of at least qualitative predictions. We ordered this LCS (and every other LCS used in this study) from a distributor and not from the manufacturer directly.

The manufacturer SPEC Sensors offers individual calibrations of sensors from the same production batch. Considering the inter-sensor unit variability for the SPEC DGS-O3, it would be interesting to see if calibrated sensors from the same batch show a reduced inter-sensor variability and measurement error. The inter-sensor variability increases the challenge of calibrating the sensors. It hints at the possible difficulty of finding a generalized calibration model for the SPEC DGS-O3. The PM LCS called SPS30 shows very good calibration performance, given the reasonably small errors during the training and testing periods. As a result, we consider this sensor to be a good choice for any future AELCM unit.

## Figures and Tables

**Table 1:** Overview of the specifications of the sensors that can be used in the AELCM unit.

Measured Variable	Sensor	Manufacturer	Measuring Range	Accuracy * (Repeatability) * [Precision] *	Approx. Price (Euro) 2020
Temperature Humidity	BME280 [30]	Bosch	−40–65 °C 0–100%	± 0.5–± 1.5 °C ±3% ^1^	5
O_3_	MQ131 [31]	Winsen	0.01–1 ppm	/	20
NO_2_	MiCS-2714 [32]	SGX Sensortech	0.05–10 ppm	/	10
CO	MiCS-4514 [33]	SGX Sensortech	1–1000 ppm	/	14
O_3_	DGS-O3 968-042 [34]	SPEC Sensors	0–5 ppm	±15% (<±3%)	80
NO_2_	DGS-NO2 968-043 [35]	SPEC Sensors	0–5 ppm	±15% (<±3%)	80
CO	DGS-CO 968-034 [36]	SPEC Sensors	0–1000 ppm	±15% (<±3%)	80
PM_1/2.5_	SPS30 [37,38]	Sensirion	0–1000 μg/m^3^	[±10 µg/m^3^ at 0 to 100 µg/m^3^] [±10% at 100 to 1000 µg/m^3^]	32

* This information is given if the manufacturer’s sensor data sheet provided it. ^1^ 20–80%RH, 25 °C, including hysteresis.

**Table 2:** General information about the deployed AELCM units’ individual LCS configurations including the amount of available data for the first atmospheric exposure monitoring network (AEMN) experiment for every deployed AELCM unit since their individual deployment dates.

No. AELCM Unit	Deployment Date	Missing Sensors *	Available Data Logger/Database
003	26 February 2021	MiCS-2714	100%/95.56%
		MiCS-4514	
004	4 June 2021	DGS-CO	100%/94.95%
	21 June 2021		
005	4 June 2021	DGS-CO	n. A./96.91%

* The standard sensor equipment of the first version of an AELCM unit is shown in Table 1. Therefore, missing sensors relate to the introduced sensors in Table 1.

**Table 3:** Statistics based on the hourly (15 min) means of the different atmospheric variables measured by the AEMS between the 27 February 2021 and 24 October 2021. For O_3_, NO_2_ and CO hourly gas [ppb] and for PM 15 min concentration means [µg/m^3^] were used. The temperature [°C] and relative humidity [%] statistics are based on hourly means.

Atmospheric Variable	Min	25th Percentile	Mean	75th Percentile	Max
O_3_	0.00	16.79	27.79	38.01	85.65
NO_2_	0.38	2.59	6.24	8.39	36.99
CO	93.44	141.55	176.89	196.69	1366.96
PM1	0.20	3.21	7.46	10.36	44.23
PM2.5	0.32	4.14	8.72	11.91	136.51
Temperature	−4.27	8.21	12.94	17.46	31.68
Relative Humidity	17.94	58.54	71.15	86.22	95.46

**Table 4:** Evaluation statistics for every LCS used in the AELCM units: Spearman rank correlation (Rs), root-mean-square error (RMSE) and the coefficient of determination (R^2^). For the calculation of R^2^ and RMSE, all model-adjusted LCS data (hourly gas [ppb] and 15 min PM concentration means [µg/m^3^]), while for Rs all raw LCS data for every individual sensor are used (excluding the data taken during the warm-up duration). The AEMS data are used as reference. For all LCS data, besides the SPS30, the RMSE is in ppb. For SPS30 data, the RMSE is in µg/m^3^.

No. AELCM Unit	DGS-O3	DGS-NO2	DGS-CO	MQ131 (O_3_)	MiCS-2714 (NO_2_)	MiCS-4514 (CO)	SPS30 (PM1/PM2.5)
003	Rs: 0.90	Rs: 0.18	Rs: −0.25	Rs: −0.55	/	/	Rs: 0.97/0.94
	RMSE: 6.74	RMSE: 3.47	RMSE: 53.67	RMSE: 6.53	/	/	RMSE: 1.07/1.96
	R^2^: 0.80	R^2^: 0.59	R^2^: 0.20	R^2^: 0.81	/	/	R^2^: 0.96/0.90
004	Rs: 0.50	Rs: −0.02	/	Rs: −0.26	Rs: 0.28	Rs: 0.39	Rs: 0.97/0.95
	RMSE: 7.79	RMSE: 3.61	/	RMSE: 7.81	RMSE: 4.16	RMSE: 30.53	RMSE: 0.77/1.27
	R^2^: 0.71	R^2^: 0.35	/	R^2^: 0.71	R^2^: 0.15	R^2^: 0.66	R^2^: 0.97/0.94
005	Rs: 0.97	Rs: −0.01	/	Rs: −0.28	Rs: 0.53	Rs: −0.49	Rs: 0.96/0.94
	RMSE: 3.31	RMSE: 3.80	/	RMSE: 6.31	RMSE: 3.51	RMSE: 44.95	RMSE: 0.87/1.51
	R^2^: 0.95	R^2^: 0.28	/	R^2^: 0.83	R^2^: 0.40	R^2^: 0.27	R^2^: 0.96/0.91

## Data Availability

The study data are available upon request from the corresponding authors.

## Supplementary Materials

The following supporting information can be downloaded at https://www.mdpi.com/article/10.3390/s22103830/s1, Figure S1.1: Top view of the AELCM sensor board with MiCS sensor sockets for the MiCS-4514 on the left and MiCS-2714 on the right side at the bottom of the printed circuit board used in this study. Figure S1.2: Side view of the AELCM sensor board with the MiCS sensor sockets for the MiCS-4514 on the left and MiCS-2714 on the right side used in this study. Figure S1.3: Top view of the latest MiCS sensor socket board for the MiCS-2714 on the left and MiCS-4514 on the right side at the top of the printed circuit board. Figure S1.4: Side view of the latest MiCS sensor socket board for the MiCS-2714 and MiCS-4514. Figure S2.1: Autocorrelation coefficients for different lags of different air pollutants measured by the Atmospheric Exposure Monitoring Station (AEMS). The observation period is between the 27 February 2021 and 24 October 2021. The concentrations of the air pollutants ozone, nitrogen dioxide and carbon monoxide are measured in the measurement unit ppb (hourly means), while the particulate matter measurements are measured in the measurement unit µg/m^3^ (15-min means). From top to bottom: O_3_, NO_2_, CO, PM2.5 and PM1. Table S3.1: Average, minimum and maximum RMSE for ambient ozone in parts per billion (ppb) and for each p predictor model regarding LCS SPEC DGS-O3 deployed with specific AELCM units. The performance estimation method is the repeated holdout procedure using reference data of the AEMS and model-calibrated (adjusted) LCS data based on hourly averages. Table S3.2: Average, minimum and maximum RMSE for ambient nitrogen dioxide in parts per billion (ppb) and for each p predictor model regarding LCS SPEC DGS-NO2 deployed with specific AELCM units. The performance estimation method is the repeated holdout procedure using reference data of the AEMS and model-calibrated (adjusted) LCS data based on hourly averages. Table S3.3: Average, minimum and maximum RMSE for ambient carbon monoxide in parts per billion (ppb) and for each p predictor model regarding LCS SPEC DGS-CO deployed with specific AELCM units. The performance estimation method is the repeated holdout procedure using reference data of the AEMS and model-calibrated (adjusted) LCS data based on hourly averages. Table S3.4: Average, minimum and maximum RMSE for ambient ozone in parts per billion (ppb) and for each p predictor model regarding LCS MQ131 deployed with specific AELCM units. The performance estimation method is the repeated holdout procedure using reference data of the AEMS and model-calibrated (adjusted) LCS data based on hourly averages. Table S3.5: Average, minimum and maximum RMSE for ambient nitrogen dioxide in parts per billion (ppb) and for each p predictor model regarding LCS SGX MiCS-2714 deployed with specific AELCM units. The performance estimation method is the repeated holdout procedure using reference data of the AEMS and model-calibrated (adjusted) LCS data based on hourly averages. Table S3.6: Average, minimum and maximum RMSE for ambient carbon monoxide in parts per billion (ppb) and for each p predictor model regarding LCS SGX MiCS-4514 deployed with specific AELCM units. The performance estimation method is the repeated holdout procedure using reference data of the AEMS and model-calibrated (adjusted) LCS data based on hourly averages. Table S3.7: Average, minimum and maximum RMSE for ambient particulate matter PM2.5 in microgram per cubic meter (µg/m^3^) and for each p predictor model regarding SPS30 deployed with specific AELCM units. The performance estimation method is the repeated holdout procedure using reference data of the AEMS and model-calibrated (adjusted) LCS data based on 15-min averages. Table S3.8: Average, minimum and maximum RMSE for ambient particulate matter PM1 in microgram per cubic meter (µg/m^3^) and for each p predictor model regarding SPS30 deployed with specific AELCM units. The performance estimation method is the repeated holdout procedure using reference data of the AEMS and model-calibrated (adjusted) LCS data based on 15-min averages. Table S4.1: Average, minimum and maximum RMSE for ambient ozone in parts per billion (ppb) and for each p predictor model regarding LCS SPEC DGS-O3 deployed with specific AELCM units. The performance estimation method is the 5-fold cross validation procedure using reference data of the AEMS and model-calibrated (adjusted) LCS data based on hourly averages. Table S4.2: Average, minimum and maximum RMSE for ambient nitrogen dioxide in parts per billion (ppb) and for each p predictor model regarding LCS SPEC DGS-NO2 deployed with specific AELCM units. The performance estimation method is the 5-fold cross validation procedure using reference data of the AEMS and model-calibrated (adjusted) LCS data based on hourly averages. Table S4.3: Average, minimum and maximum RMSE for ambient carbon monoxide in parts per billion (ppb) and for each p predictor model regarding LCS SPEC DGS-CO deployed with specific AELCM units. The performance estimation method is the 5-fold cross validation procedure using reference data of the AEMS and model-calibrated (adjusted) LCS data based on hourly averages. Table S4.4: Average, minimum and maximum RMSE for ambient ozone in parts per billion (ppb) and for each p predictor model regarding LCS MQ131 deployed with specific AELCM units. The performance estimation method is the 5-fold cross validation procedure using reference data of the AEMS and model-calibrated (adjusted) LCS data based on hourly averages. Table S4.5: Average, minimum and maximum RMSE for ambient nitrogen dioxide in parts per billion (ppb) and for each p predictor model regarding LCS SGX MiCS-2714 deployed with specific AELCM units. The performance estimation method is the 5-fold cross validation procedure using reference data of the AEMS and model-calibrated (adjusted) LCS data based on hourly averages. Table S4.6: Average, minimum and maximum RMSE for ambient carbon monoxide in parts per billion (ppb) and for each p predictor model regarding LCS SGX MiCS-4514 deployed with specific AELCM units. The performance estimation method is the 5-fold cross validation procedure using reference data of the AEMS and model-calibrated (adjusted) LCS data based on hourly averages. Table S4.7: Average, minimum and maximum RMSE for ambient particulate matter PM2.5 in microgram per cubic meter (µg/m^3^) and for each p predictor model regarding SPS30 deployed with specific AELCM units. The performance estimation method is the 5-fold cross validation procedure using reference data of the AEMS and model-calibrated (adjusted) LCS data based on 15-min averages. Table S4.8: Average, minimum and maximum RMSE for ambient particulate matter PM1 in microgram per cubic meter (µg/m^3^) and for each p predictor model regarding SPS30 deployed with specific AELCM units. The performance estimation method is the 5-fold cross validation procedure using reference data of the AEMS and model-calibrated (adjusted) LCS data based on 15-min averages. Table S5.1: Spearman rank correlations between different LCS output (MQ131 output, temperature and relative humidity) and reference measurements of ambient ozone for all AELCM units. As starting points for AELCM003, AELCM004 and AELCM005 we use the measured data at 5 March 2021, 28 June 2021 and 11 June 2021 respectively. The ending date is the 24 October 2021. Figure S6.1: Model-adjusted hourly concentration means of different deployed SPEC DGS-NO2 using multiple linear regression vs. hourly concentration means of reference measurements given by the AEMS for ambient nitrogen dioxide in ppb. For smoothing the hourly concentration means a rolling 24-h average is used: (Top) SPEC DGS-NO2 deployed in AELCM003; (Middle) SPEC DGS-NO2 deployed in AELCM004; (Bottom) SPEC DGS-NO2 deployed in AELCM005. Figure S6.2: Model-adjusted hourly concentration means of a SPEC DGS-CO deployed in AELCM003 using multiple linear regression vs. hourly concentration means of reference measurements given by the AEMS for ambient carbon monoxide in ppb. For smoothing the hourly concentration means a rolling 24-h average is used. Figure S6.3: Model-adjusted hourly concentration means of different deployed MQ131 using multiple linear regression vs. hourly concentration means of reference measurements given by the AEMS for ambient ozone in ppb. For smoothing the hourly concentration means a rolling 24-h average is used: (Top) MQ131 deployed in AELCM003; (Middle) MQ131 deployed in AELCM004; (Bottom) MQ131 deployed in AELCM005. Figure S6.4: Model-adjusted hourly concentration means of different deployed MiCS-2714 using multiple linear regression vs. hourly concentration means of reference measurements given by the AEMS for ambient nitrogen dioxide in ppb. For smoothing the hourly concentration means a rolling 24-h average is used: (Top) MiCS-2714 deployed in AELCM004; (Bottom) MiCS-2714 deployed in AELCM005. Figure S6.5: Model-adjusted hourly concentration means of different deployed MiCS-4514 using multiple linear regression vs. hourly concentration means of reference measurements given by the AEMS for ambient carbon monoxide in ppb. For smoothing the hourly concentration means a rolling 24-h average is used: (Top) MiCS-4514 deployed in AELCM004; (Bottom) MiCS-4514 deployed in AELCM005. Figure S7.1: Raw hourly means of ambient ozone measured by SPEC DGS-O3 units deployed in AELCM003, AELCM004 and AELCM005 compared with hourly means of ambient ozone measurements given by the reference station AEMS. For smoothing the hourly concentration means a rolling 24-h average is used.
