# Supplementary material for: Development of Air Quality Boxes Based on Low-Cost Sensor Technology for Ambient Air Quality Monitoring
Source: Sensors (Basel). 2022 May 18;22(10):3830. doi: 10.3390/s22103830 (PMC9144299; doi:10.3390/s22103830)
Supplement: Supplementary file 1 [file sensors-22-03830-s001.zip › sensors-1670136-supplementary.pdf]

S 1: Custom SMD sockets and carrier board for SGX Sensortech MiCS sensors

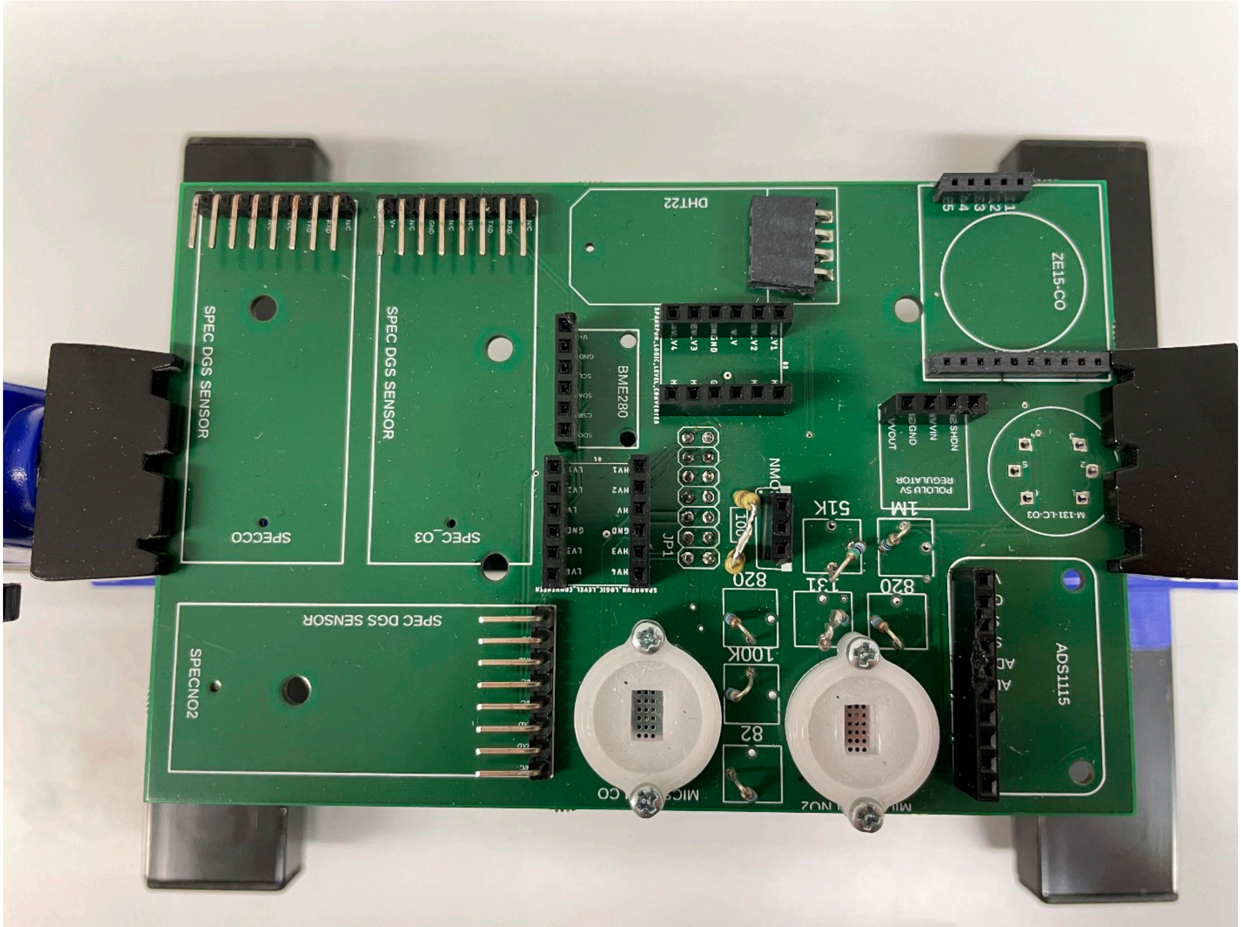

Figure S1.1. Top view of the AELCM sensor board with MiCS sensor sockets for the MiCS-4514 on the left and MiCS-2714 on the right side at the bottom of the printed circuit board used in this study.

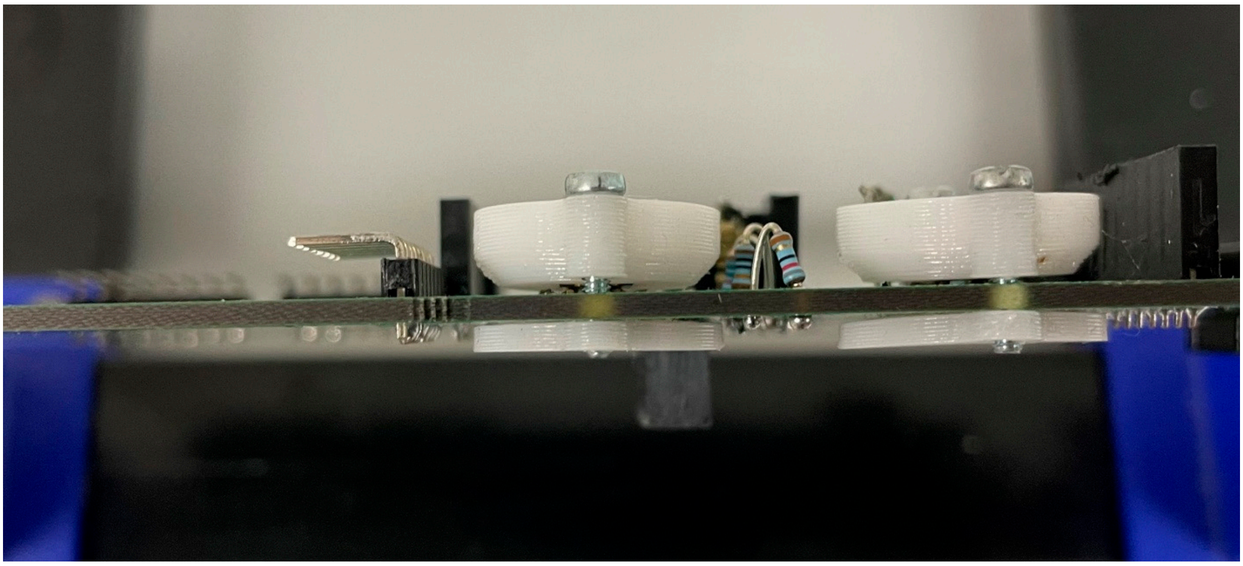

Figure S1.2. Side view of the AELCM sensor board with the MiCS sensor sockets for the MiCS-4514 on the left and MiCS-2714 on the right side used in this study.

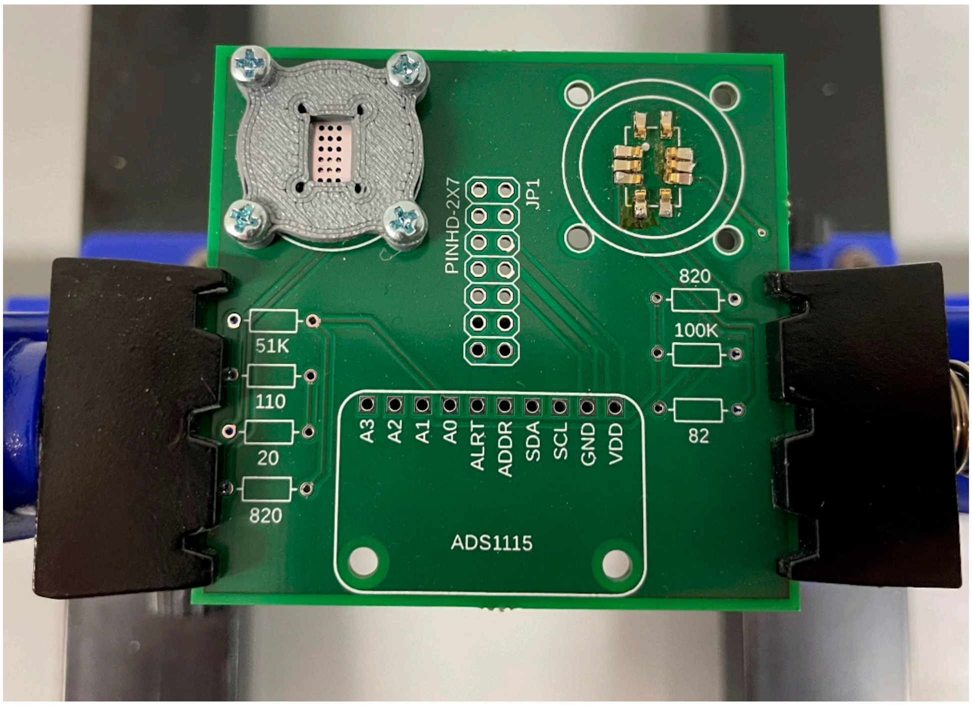

Figure S1.3. Top view of the latest MiCS sensor socket board for the MiCS-2714 on the left and MiCS-4514 on the right side at the top of the printed circuit board.

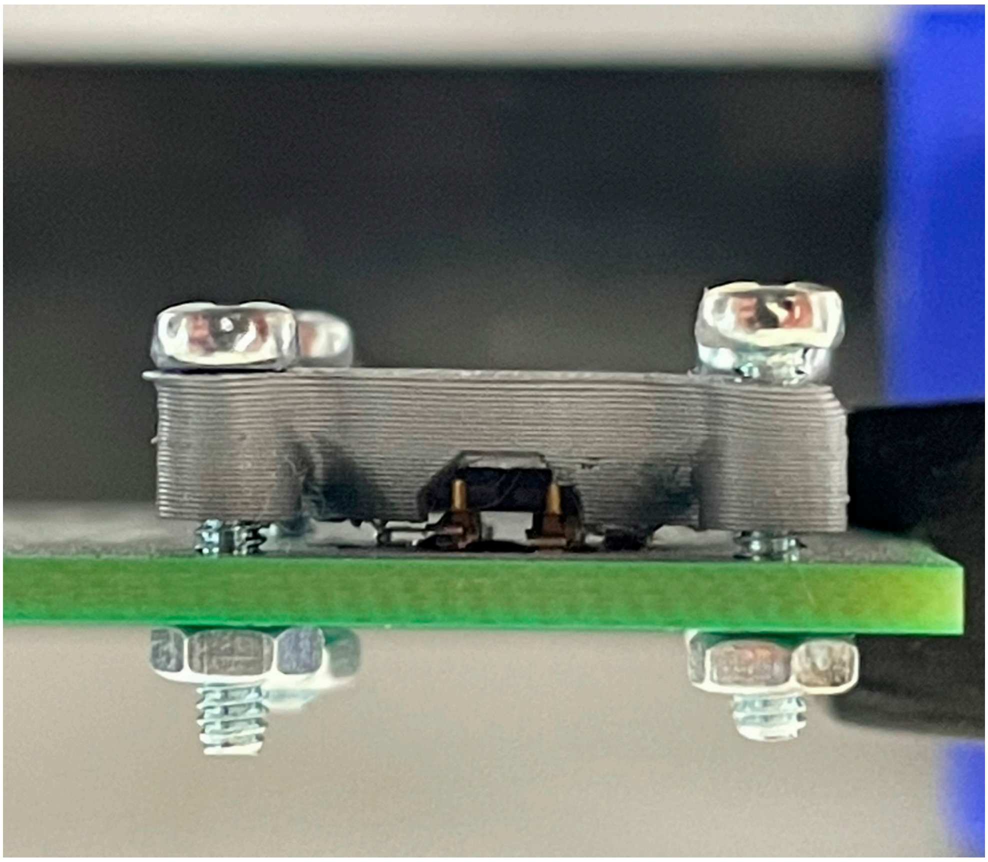

Figure S1.4. Side view of the latest MiCS sensor socket board for the MiCS-2714 and MiCS-4514.

S 2: Autocorrelation of Station Observations (AEMS)

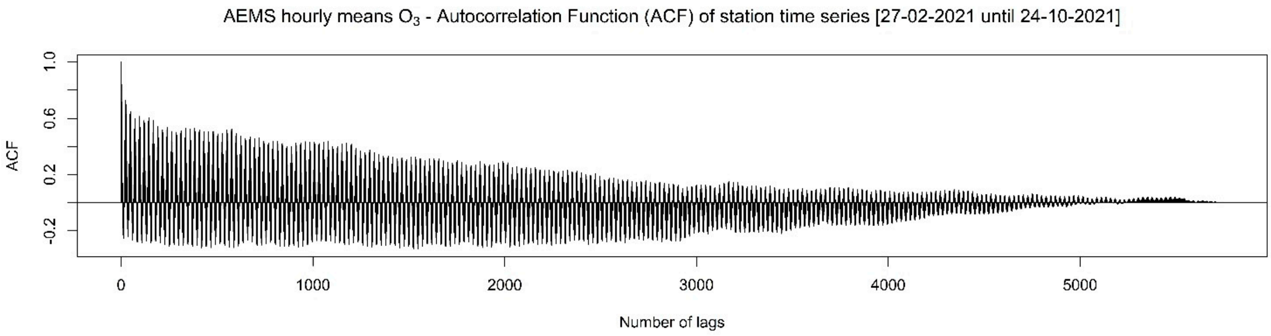

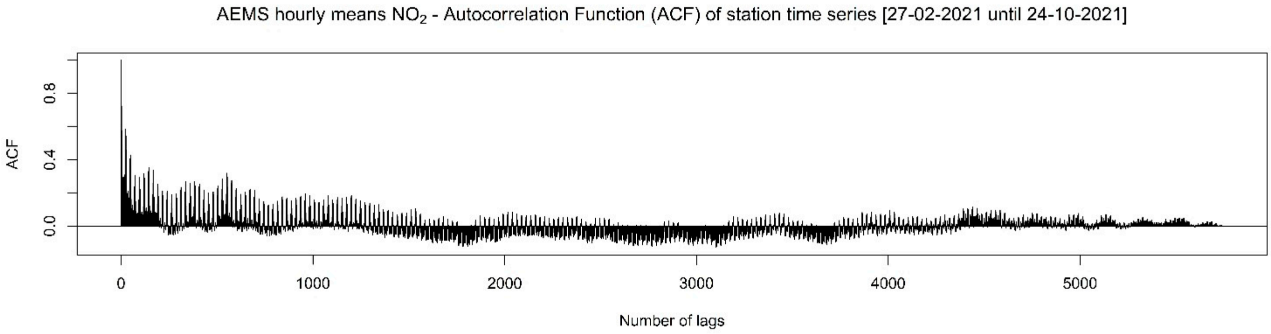

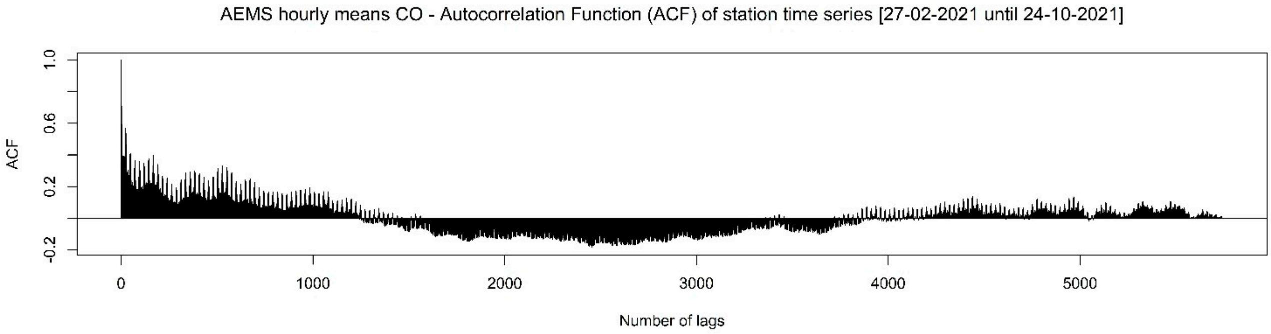

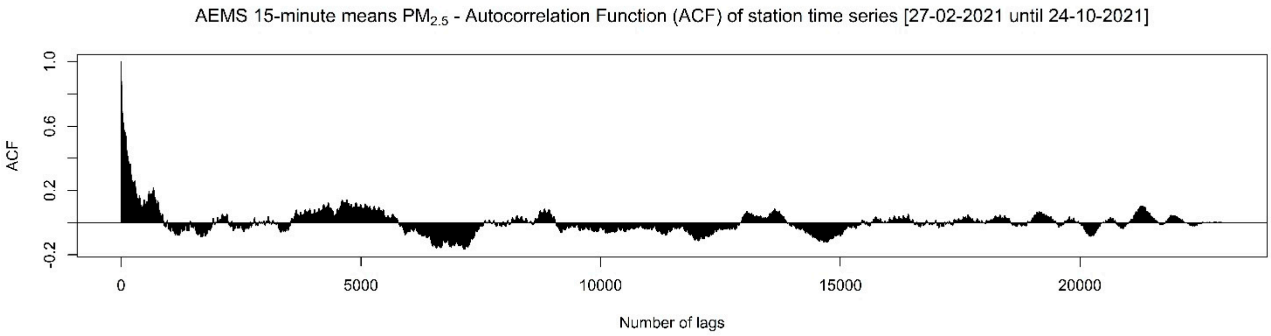

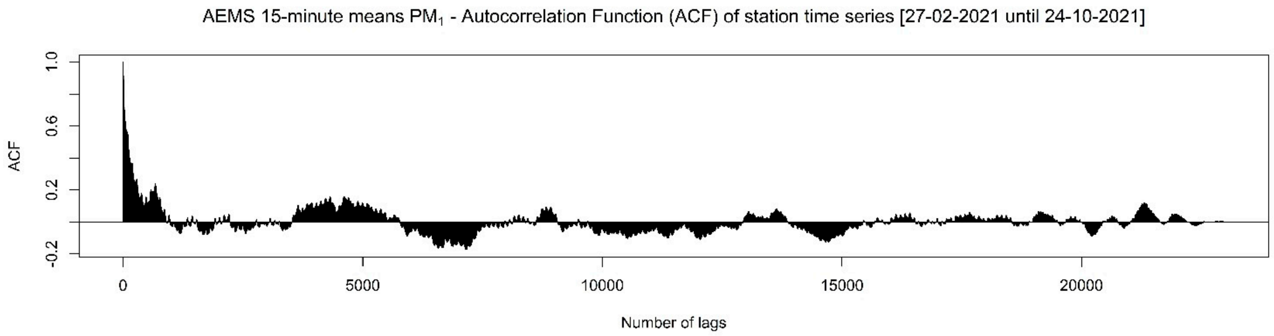

Figure S2.1. Autocorrelation coefficients for different lags of different air pollutants measured by the Atmospheric Exposure Monitoring Station (AEMS). The observation period is between the 27-02-2021 and 24-10-2021. The concentrations of the air pollutants ozone, nitrogen dioxide and carbon monoxide are measured in the measurement unit ppb (hourly means), while the particulate matter measurements are measured in the measurement unit μg/m^3 (15-minute means). From top to bottom: O_3, NO_2, CO, PM2.5 and PM1.

S 3: Stepwise Regression (OOS)

Table S3.1. Average, minimum and maximum RMSE for ambient ozone in parts per billion (ppb) and for each p predictor model regarding LCS SPEC DGS-O3 deployed with specific AELCM units. The performance estimation method is the repeated holdout procedure using reference data of the AEMS and model-calibrated (adjusted) LCS data based on hourly averages.

No. AELCM unit	Number of predictors	Training Avg. RMSE	Training Min. RMSE	Training Max. RMSE	Evaluation Avg. RMSE	Evaluation Min. RMSE	Evaluation Max. RMSE
003	1	7.74	7.64	7.87	9.41	7.72	10.52
	2	6.47	6.30	6.64	8.37	6.60	9.88
	3	6.35	6.18	6.56	8.32	5.89	10.33
	4	6.34	6.17	6.55	8.21	5.93	9.90
004	1	8.45	8.14	8.62	7.99	6.61	9.17
	2	8.04	7.72	8.25	7.02	5.48	9.15
	3	8.36	7.63	8.99	8.05	5.36	10.93
	4	7.95	7.63	8.18	6.88	5.35	9.30
005	1	3.79	3.18	4.20	3.41	2.73	3.80
	2	3.37	2.82	3.82	3.17	2.59	3.49
	3	3.39	2.86	3.83	2.95	2.09	3.64
	4	3.09	2.49	3.65	2.67	2.42	3.09

Table S3.2. Average, minimum and maximum RMSE for ambient nitrogen dioxide in parts per billion (ppb) and for each p predictor model regarding LCS SPEC DGS-NO2 deployed with specific AELCM units. The performance estimation method is the repeated holdout procedure using reference data of the AEMS and model-calibrated (adjusted) LCS data based on hourly averages.

No. AELCM unit	Number of predictors	Training Avg. RMSE	Training Min. RMSE	Training Max. RMSE	Evaluation Avg. RMSE	Evaluation Min. RMSE	Evaluation Max. RMSE
003	1	4.33	4.01	4.51	4.55	3.39	6.22
	2	3.29	3.08	3.70	3.90	3.23	4.93
	3	3.05	2.86	3.32	3.47	2.98	4.44
004	1	4.19	3.90	4.37	4.51	3.84	5.00
	2	3.70	3.52	3.84	3.97	3.58	4.30
	3	3.61	3.47	3.74	3.94	3.56	4.27
005	1	3.94	3.47	4.29	4.91	3.91	6.78
	2	3.70	3.41	3.91	4.66	3.89	6.32
	3	3.62	3.41	3.87	4.25	3.37	6.29

Table S3.3. Average, minimum and maximum RMSE for ambient carbon monoxide in parts per billion (ppb) and for each p predictor model regarding LCS SPEC DGS-CO deployed with specific AELCM units. The performance estimation method is the repeated holdout procedure using reference data of the AEMS and model-calibrated (adjusted) LCS data based on hourly averages.

No. AELCM Unit	Number of predictors	Training Avg. RMSE	Training Min. RMSE	Training Max. RMSE	Evaluation Avg. RMSE	Evaluation Min. RMSE	Evaluation Max. RMSE
003	1	44.67	41.15	55.83	50.87	38.15	72.06
	2	44.13	40.84	54.79	52.73	42.03	66.91
	3	44.04	40.78	54.79	52.41	42.18	66.09

Table S3.4. Average, minimum and maximum RMSE for ambient ozone in parts per billion (ppb) and for each p predictor model regarding LCS MQ131 deployed with specific AELCM units. The performance estimation method is the repeated holdout procedure using reference data of the AEMS and model-calibrated (adjusted) LCS data based on hourly averages.

No. AELCM Unit	Number of predictors	Training Avg. RMSE	Training Min. RMSE	Training Max. RMSE	Evaluation Avg. RMSE	Evaluation Min. RMSE	Evaluation Max. RMSE
003	1	9.30	8.73	9.59	8.55	7.92	9.05
	2	8.60	8.20	9.08	10.09	5.14	12.56
	3	6.83	6.56	7.14	7.07	4.50	8.53
004	1	8.59	8.43	8.71	7.76	6.47	9.10
	2	8.12	8.03	8.19	7.14	6.07	8.63
	3	8.10	8.01	8.18	7.17	6.06	8.76
005	1	8.08	8.00	8.51	7.90	6.85	11.07
	2	5.89	5.68	7.01	7.34	4.91	8.70
	3	5.64	5.39	6.70	5.23	3.90	6.32

Table S3.5. Average, minimum and maximum RMSE for ambient nitrogen dioxide in parts per billion (ppb) and for each p predictor model regarding LCS SGX MiCS-2714 deployed with specific AELCM units. The performance estimation method is the repeated holdout procedure using reference data of the AEMS and model-calibrated (adjusted) LCS data based on hourly averages.

No. AELCM unit	Number of predictors	Training Avg. RMSE	Training Min. RMSE	Training Max. RMSE	Evaluation Avg. RMSE	Evaluation Min. RMSE	Evaluation Max. RMSE
004	1	4.37	4.10	4.47	4.25	3.41	5.26
	2	4.21	3.98	4.33	4.03	3.27	4.67
	3	4.21	3.96	4.33	4.03	3.34	4.61
005	1	3.51	2.94	3.83	4.57	3.89	5.92
	2	3.48	2.93	3.77	4.29	3.32	5.94
	3	3.37	2.80	3.67	4.15	3.23	5.82

Table S3.6. Average, minimum and maximum RMSE for ambient carbon monoxide in parts per billion (ppb) and for each p predictor model regarding LCS SGX MiCS-4514 deployed with specific AELCM units. The performance estimation method is the repeated holdout procedure using reference data of the AEMS and model-calibrated (adjusted) LCS data based on hourly averages.

No. AELCM unit	Number of predictors	Training Avg. RMSE	Training Min. RMSE	Training Max. RMSE	Evaluation Avg. RMSE	Evaluation Min. RMSE	Evaluation Max. RMSE
004	1	31.68	29.63	34.28	64.29	46.51	86.86
	2	29.61	28.16	31.06	53.41	38.77	68.43
	3	26.60	25.38	27.50	40.04	30.28	49.94
005	1	38.48	33.28	41.20	55.96	43.03	68.44
	2	37.95	33.04	41.14	54.68	41.37	67.89
	3	37.93	33.04	40.89	54.85	41.42	66.56

Table S3.7. Average, minimum and maximum RMSE for ambient particulate matter PM2.5 in microgram per cubic meter (μg/m^3) and for each p predictor model regarding SPS30 deployed with specific AELCM units. The performance estimation method is the repeated holdout procedure using reference data of the AEMS and model-calibrated (adjusted) LCS data based on 15-minute averages.

No. AELCM Unit	Number of predictors	Training Avg. RMSE	Training Min. RMSE	Training Max. RMSE	Evaluation Avg. RMSE	Evaluation Min. RMSE	Evaluation Max. RMSE
003	1	2.42	1.99	2.62	1.81	1.61	2.10
	2	1.97	1.53	2.15	1.66	1.01	2.12
	3	1.85	1.46	2.08	1.50	1.13	1.77
	4	1.87	1.50	2.08	1.53	1.06	1.79
004	1	1.68	1.59	1.72	1.67	1.34	1.88
	2	1.25	1.18	1.31	1.25	1.18	1.43
	3	1.23	1.16	1.29	1.23	1.04	1.92
	4	1.21	1.15	1.26	1.30	0.96	2.46
005	1	1.89	1.67	2.21	1.67	1.48	1.98
	2	1.43	1.26	1.68	1.29	1.09	1.48
	3	1.37	1.20	1.59	1.43	1.10	1.79
	4	1.38	1.20	1.61	1.38	1.15	1.92

Table S3.8. Average, minimum and maximum RMSE for ambient particulate matter PM1 in microgram per cubic meter (μg/m^3) and for each p predictor model regarding SPS30 deployed with specific AELCM units. The performance estimation method is the repeated holdout procedure using reference data of the AEMS and model-calibrated (adjusted) LCS data based on 15-minute averages.

No. AELCM Unit	Number of predictors	Training Avg. RMSE	Training Min. RMSE	Training Max. RMSE	Evaluation Avg. RMSE	Evaluation Min. RMSE	Evaluation Max. RMSE
003	1	1.59	1.41	1.69	1.51	1.15	2.04
	2	1.05	0.86	1.14	1.18	0.92	1.36
	3	0.94	0.83	1.03	1.01	0.73	1.09
	4	0.92	0.84	1.03	1.04	0.81	1.12
004	1	1.30	1.28	1.33	1.19	1.02	1.37
	2	0.79	0.77	0.81	0.70	0.53	0.83
	3	0.85	0.76	0.96	0.94	0.50	1.80
	4	0.79	0.77	0.82	0.75	0.58	0.89

005	1	1.35	1.27	1.47	1.40	1.14	1.57
	2	0.87	0.85	0.89	0.88	0.67	1.20
	3	0.92	0.84	1.01	1.10	0.78	1.98
	4	0.86	0.85	0.89	0.90	0.75	1.23

S 4: Stepwise Regression (CV)

Table S4.1. Average, minimum and maximum RMSE for ambient ozone in parts per billion (ppb) and for each p predictor model regarding LCS SPEC DGS-O3 deployed with specific AELCM units. The performance estimation method is the 5-fold cross validation procedure using reference data of the AEMS and model-calibrated (adjusted) LCS data based on hourly averages.

No. AELCM unit	Number of predictors	Training Avg. RMSE	Training Min. RMSE	Training Max. RMSE	Evaluation Avg. RMSE	Evaluation Min. RMSE	Evaluation Max. RMSE
003	1	8.14	7.89	8.48	8.80	7.09	10.18
	2	6.86	6.45	7.17	7.40	5.89	9.33
	3	6.67	6.34	6.90	7.52	6.32	9.31
	4	6.64	6.34	6.85	7.51	6.41	9.51
004	1	8.47	8.23	8.68	8.86	7.81	9.70
	2	7.84	7.61	8.15	8.02	6.73	8.93
	3	9.15	8.10	9.61	8.86	6.61	11.25
	4	7.76	7.56	8.10	8.04	6.61	8.78
005	1	3.90	3.29	4.07	4.04	3.41	6.21
	2	3.53	2.95	3.71	3.67	3.01	5.71
	3	3.58	2.96	3.83	3.85	2.81	5.80
	4	3.27	2.59	3.49	3.36	2.63	5.59

Table S4.2. Average, minimum and maximum RMSE for ambient nitrogen dioxide in parts per billion (ppb) and for each p predictor model regarding LCS SPEC DGS-NO2 deployed with specific AELCM units. The performance estimation method is the 5-fold cross validation procedure using reference data of the AEMS and model-calibrated (adjusted) LCS data based on hourly averages.

No. AELCM unit	Number of predictors	Training Avg. RMSE	Training Min. RMSE	Training Max. RMSE	Evaluation Avg. RMSE	Evaluation Min. RMSE	Evaluation Max. RMSE
003	1	4.84	4.38	5.12	4.76	3.49	6.24
	2	3.72	3.24	3.98	3.81	3.07	5.24
	3	3.43	3.10	3.63	3.73	3.00	4.80
004	1	4.10	3.70	4.35	4.11	3.08	5.55
	2	3.68	3.40	3.87	3.77	3.00	4.82
	3	3.60	3.37	3.74	3.73	3.20	4.64
005	1	4.11	3.69	4.35	4.17	3.19	5.72
	2	3.89	3.53	4.15	3.99	2.79	5.33
	3	3.76	3.51	3.99	4.05	3.00	5.10

Table S4.3. Average, minimum and maximum RMSE for ambient carbon monoxide in parts per billion (ppb) and for each p predictor model regarding LCS SPEC DGS-CO deployed with specific AELCM units. The performance estimation method is the 5-fold cross validation procedure using reference data of the AEMS and model-calibrated (adjusted) LCS data based on hourly averages.

No. AELCM unit	Number of predictors	Training Avg. RMSE	Training Min. RMSE	Training Max. RMSE	Evaluation Avg. RMSE	Evaluation Min. RMSE	Evaluation Max. RMSE
003	1	53.78	48.13	57.17	54.69	40.43	73.52
	2	53.34	47.30	56.78	55.65	39.95	74.14
	3	53.28	47.16	56.76	55.17	39.84	75.04

Table S4.4. Average, minimum and maximum RMSE for ambient ozone in parts per billion (ppb) and for each p predictor model regarding LCS MQ131 deployed with specific AELCM units. The performance estimation method is the 5-fold cross validation procedure using reference data of the AEMS and model-calibrated (adjusted) LCS data based on hourly averages.

No. AELCM Unit	Number of predictors	Training Avg. RMSE	Training Min. RMSE	Training Max. RMSE	Evaluation Avg. RMSE	Evaluation Min. RMSE	Evaluation Max. RMSE
003	1	9.18	8.87	9.50	9.50	8.23	10.86
	2	8.80	8.54	9.22	10.92	8.53	14.20
	3	6.48	6.13	6.73	6.93	6.14	8.29
004	1	8.40	8.01	8.56	8.71	7.94	10.05
	2	7.80	7.48	8.09	7.95	6.77	9.13
	3	7.80	7.48	8.08	7.96	6.83	9.11
005	1	8.26	7.89	8.45	8.47	7.87	9.74
	2	7.23	6.03	7.66	9.25	6.97	14.18
	3	6.20	5.28	6.62	7.07	5.41	11.17

Table S4.5. Average, minimum and maximum RMSE for ambient nitrogen dioxide in parts per billion (ppb) and for each p predictor model regarding LCS SGX MiCS-2714 deployed with specific AELCM units. The performance estimation method is the 5-fold cross validation procedure using reference data of the AEMS and model-calibrated (adjusted) LCS data based on hourly averages.

No. AELCM unit	Number of predictors	Training Avg. RMSE	Training Min. RMSE	Training Max. RMSE	Evaluation Avg. RMSE	Evaluation Min. RMSE	Evaluation Max. RMSE
004	1	4.16	3.89	4.40	4.17	3.14	5.21
	2	4.12	3.88	4.28	6.21	3.69	13.26
	3	4.12	3.88	4.28	6.26	3.71	13.47
005	1	3.78	3.45	4.01	3.98	2.98	5.14
	2	3.61	3.22	3.81	3.76	3.01	5.09
	3	3.49	3.02	3.72	3.54	2.64	5.15

Table S4.6. Average, minimum and maximum RMSE for ambient carbon monoxide in parts per billion (ppb) and for each p predictor model regarding LCS SGX MiCS-4514 deployed with specific AELCM units. The performance

estimation method is the 5-fold cross validation procedure using reference data of the AEMS and model-calibrated (adjusted) LCS data based on hourly averages.

No. AELCM unit	Number of predictors	Training Avg. RMSE	Training Min. RMSE	Training Max. RMSE	Evaluation Avg. RMSE	Evaluation Min. RMSE	Evaluation Max. RMSE
004	1	44.49	33.79	48.62	53.22	38.73	86.36
	2	37.00	31.60	39.62	42.20	28.58	71.40
	3	30.11	27.34	31.57	33.08	25.81	46.55
005	1	45.14	39.30	47.32	48.82	35.86	74.25
	2	44.16	38.24	46.92	44.65	31.09	67.83
	3	44.09	38.24	46.92	45.06	31.22	67.82

Table S4.7. Average, minimum and maximum RMSE for ambient particulate matter PM2.5 in microgram per cubic meter (μg/m^3) and for each p predictor model regarding SPS30 deployed with specific AELCM units. The performance estimation method is the 5-fold cross validation procedure using reference data of the AEMS and model-calibrated (adjusted) LCS data based on 15-minute averages.

No. AELCM Unit	Number of predictors	Training Avg. RMSE	Training Min. RMSE	Training Max. RMSE	Evaluation Avg. RMSE	Evaluation Min. RMSE	Evaluation Max. RMSE
003	1	2.47	2.11	2.61	2.45	1.91	3.64
	2	2.00	1.67	2.16	2.02	1.41	2.98
	3	1.94	1.55	2.10	1.95	1.33	3.23
	4	1.93	1.57	2.09	1.97	1.26	3.10
004	1	1.66	1.56	1.70	1.70	1.48	2.06
	2	1.27	1.18	1.31	1.27	1.08	1.72
	3	1.40	1.28	1.46	1.52	1.20	2.11
	4	1.26	1.17	1.33	1.42	1.15	1.81
005	1	2.00	1.66	2.14	1.98	1.36	3.10
	2	1.51	1.27	1.60	1.47	0.99	2.26
	3	1.59	1.40	1.74	1.58	1.05	2.51
	4	1.49	1.27	1.59	1.58	1.06	2.30

Table S4.8. Average, minimum and maximum RMSE for ambient particulate matter PM1 in microgram per cubic meter (μg/m^3) and for each p predictor model regarding SPS30 deployed with specific AELCM units. The performance estimation method is the 5-fold cross validation procedure using reference data of the AEMS and model-calibrated (adjusted) LCS data based on 15-minute averages.

No. AELCM Unit	Number of predictors	Training Avg. RMSE	Training Min. RMSE	Training Max. RMSE	Evaluation Avg. RMSE	Evaluation Min. RMSE	Evaluation Max. RMSE
003	1	1.65	1.55	1.71	1.70	1.41	2.00
	2	1.12	1.04	1.17	1.20	0.92	1.56
	3	1.05	0.94	1.12	1.14	0.89	1.53
	4	1.05	0.92	1.12	1.18	0.89	1.60
004	1	1.24	1.20	1.28	1.28	1.09	1.40
	2	0.77	0.73	0.79	0.79	0.66	1.00
	3	0.95	0.72	1.04	1.21	0.95	1.96
	4	0.78	0.72	0.80	0.84	0.66	1.07

005	1	1.41	1.28	1.47	1.44	1.18	1.88
	2	0.87	0.85	0.91	0.90	0.75	1.02
	3	0.96	0.86	1.12	1.09	0.87	1.32
	4	0.87	0.86	0.91	0.94	0.81	1.04

S 5: Spearman rank correlation (MQ131, BME280)

Table S5.1. Spearman rank correlations between different LCS output (MQ131 output, temperature and relative humidity) and reference measurements of ambient ozone for all AELCM units. As starting points for AELCM003, AELCM004 and AELCM005 we use the measured data at 05-03-2021, 28-06-2021 and 11-06-2021 respectively. The ending date is the 24-10-2021.

No. AELCM Unit	MQ131 (Ozone)	BME280 (Temperature)	BME280 (Relative Humidity)
003	-0.55	0.47	-0.77
004	-0.26	0.71	-0.76
005	-0.28	0.74	-0.80

S 6: Model-adjusted LCS

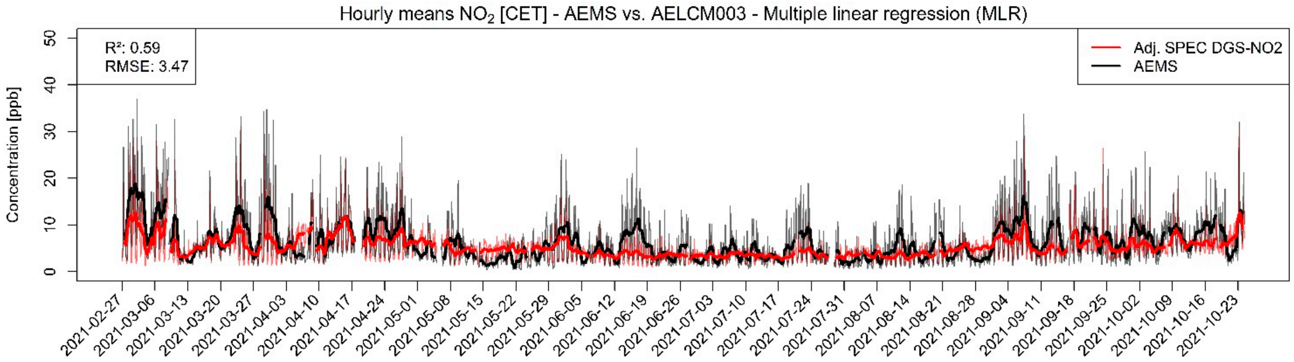

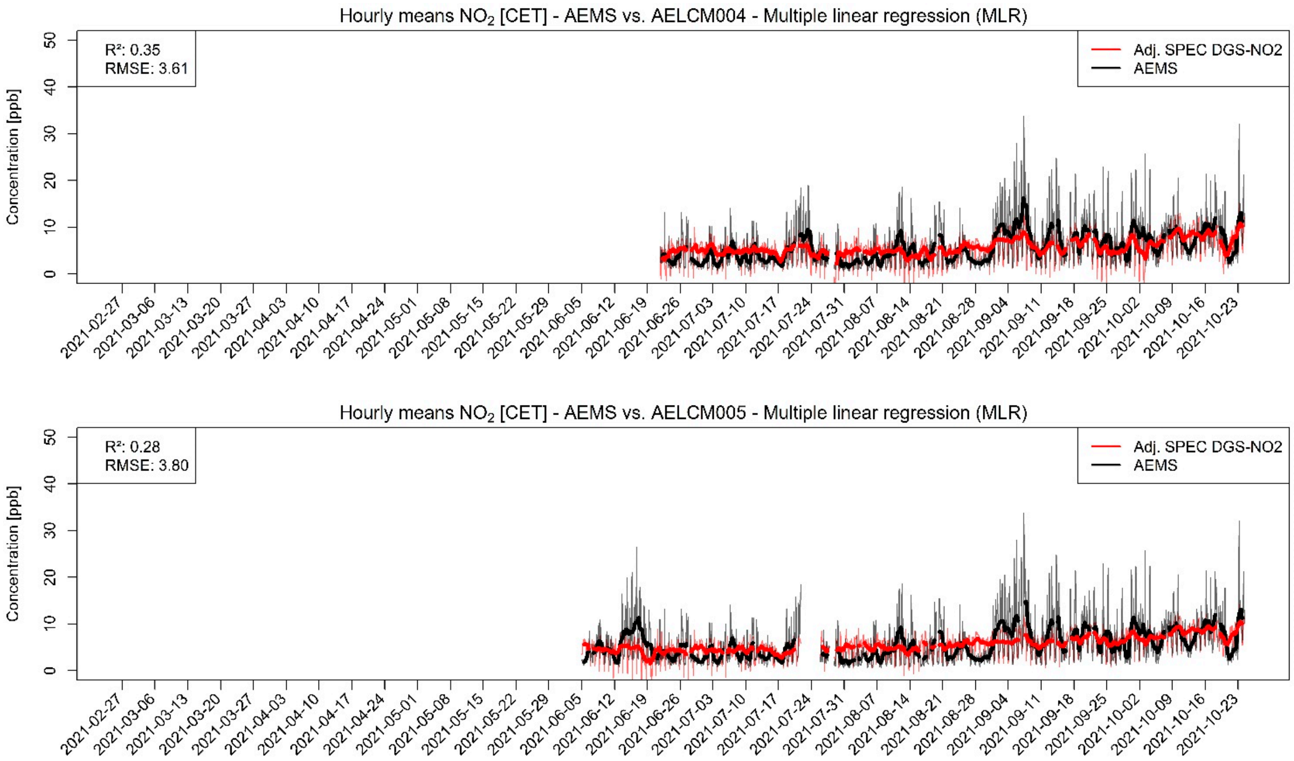

Figure S6.1. Model-adjusted hourly concentration means of different deployed SPEC DGS-NO2 using multiple linear regression vs. hourly concentration means of reference measurements given by the AEMS for ambient nitrogen dioxide in ppb. For smoothing the hourly concentration means a rolling 24-hour average is used: (**Top**) SPEC DGS-NO2 deployed in AELCM003; (**Middle**) SPEC DGS-NO2 deployed in AELCM004; (**Bottom**) SPEC DGS-NO2 deployed in AELCM005.

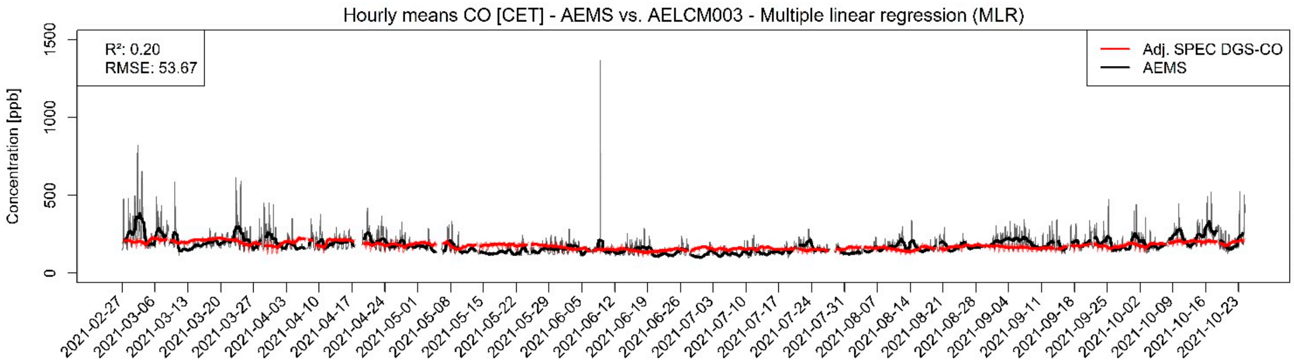

Figure S6.2. Model-adjusted hourly concentration means of a SPEC DGS-CO deployed in AELCM003 using multiple linear regression vs. hourly concentration means of reference measurements given by the AEMS for ambient carbon monoxide in ppb. For smoothing the hourly concentration means a rolling 24-hour average is used.

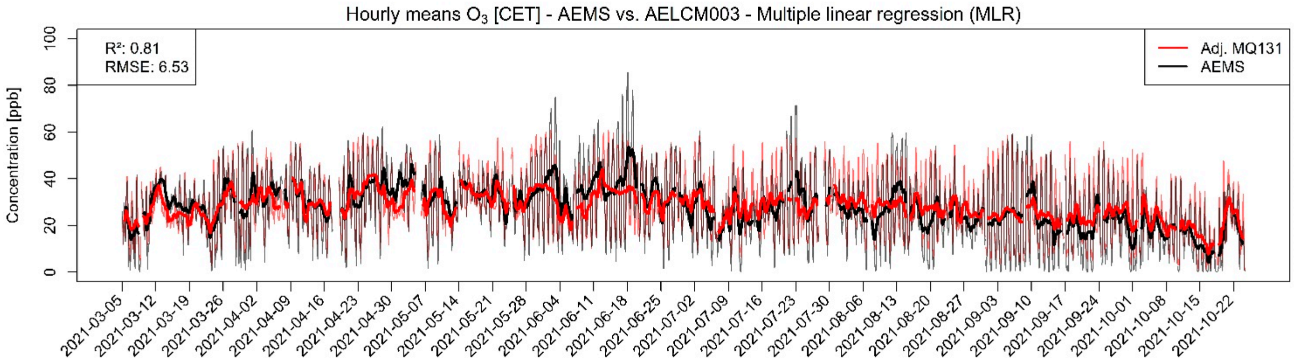
Hourly means O_3 [CET] - AEMS vs. AELCM003 - Multiple linear regression (MLR)
R²: 0.81
RMSE: 6.53
Adj. MQ131
AEMS
Concentration [ppb]
0
20
40
60
80
100
2021-03-05
2021-03-12
2021-03-19
2021-03-26
2021-04-02
2021-04-09
2021-04-16
2021-04-23
2021-04-30
2021-05-07
2021-05-14
2021-05-21
2021-05-28
2021-06-04
2021-06-11
2021-06-18
2021-06-25
2021-07-02
2021-07-09
2021-07-16
2021-07-23
2021-07-30
2021-08-06
2021-08-13
2021-08-20
2021-08-27
2021-09-03
2021-09-10
2021-09-17
2021-09-24
2021-10-01
2021-10-08
2021-10-15
2021-10-22

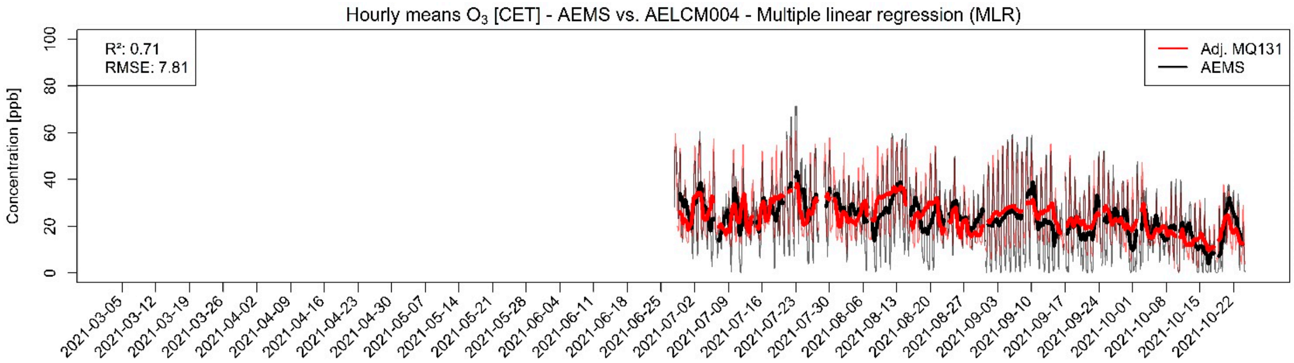
Hourly means O_3 [CET] - AEMS vs. AELCM004 - Multiple linear regression (MLR)
R²: 0.71
RMSE: 7.81
Adj. MQ131
AEMS
Concentration [ppb]
0
20
40
60
80
100
2021-03-05
2021-03-12
2021-03-19
2021-03-26
2021-04-02
2021-04-09
2021-04-16
2021-04-23
2021-04-30
2021-05-07
2021-05-14
2021-05-21
2021-05-28
2021-06-04
2021-06-11
2021-06-18
2021-06-25
2021-07-02
2021-07-09
2021-07-16
2021-07-23
2021-07-30
2021-08-06
2021-08-13
2021-08-20
2021-08-27
2021-09-03
2021-09-10
2021-09-17
2021-09-24
2021-10-01
2021-10-08
2021-10-15
2021-10-22

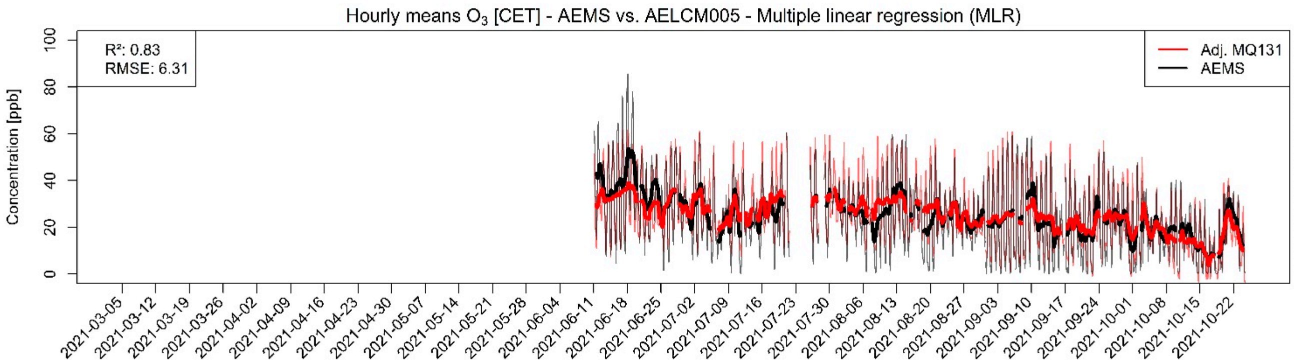

Figure S6.3. Model-adjusted hourly concentration means of different deployed MQ131 using multiple linear regression vs. hourly concentration means of reference measurements given by the AEMS for ambient ozone in ppb. For smoothing the hourly concentration means a rolling 24-hour average is used: (**Top**) MQ131 deployed in AELCM003; (**Middle**) MQ131 deployed in AELCM004; (**Bottom**) MQ131 deployed in AELCM005.

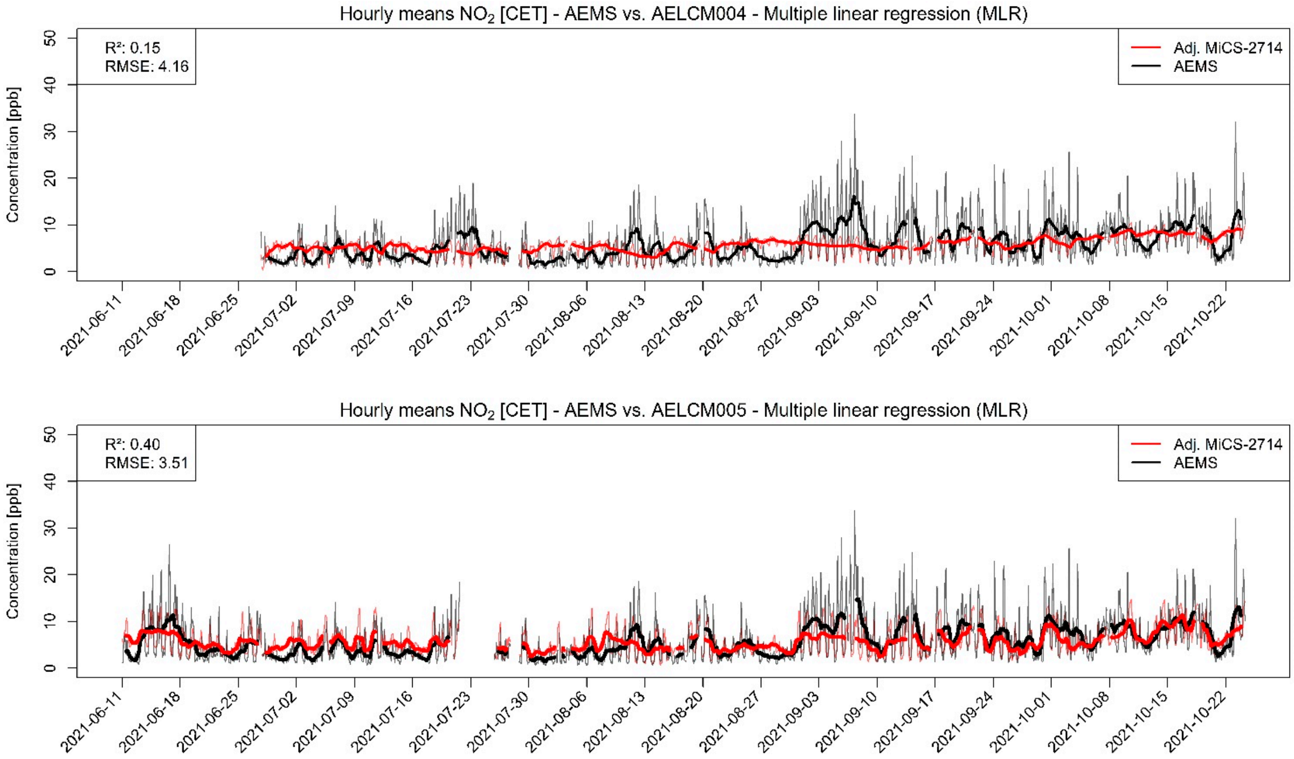

Figure S6.4. Model-adjusted hourly concentration means of different deployed MiCS-2714 using multiple linear regression vs. hourly concentration means of reference measurements given by the AEMS for ambient nitrogen dioxide in ppb. For smoothing the hourly concentration means a rolling 24-hour average is used: (**Top**) MiCS-2714 deployed in AELCM004; (**Bottom**) MiCS-2714 deployed in AELCM005.

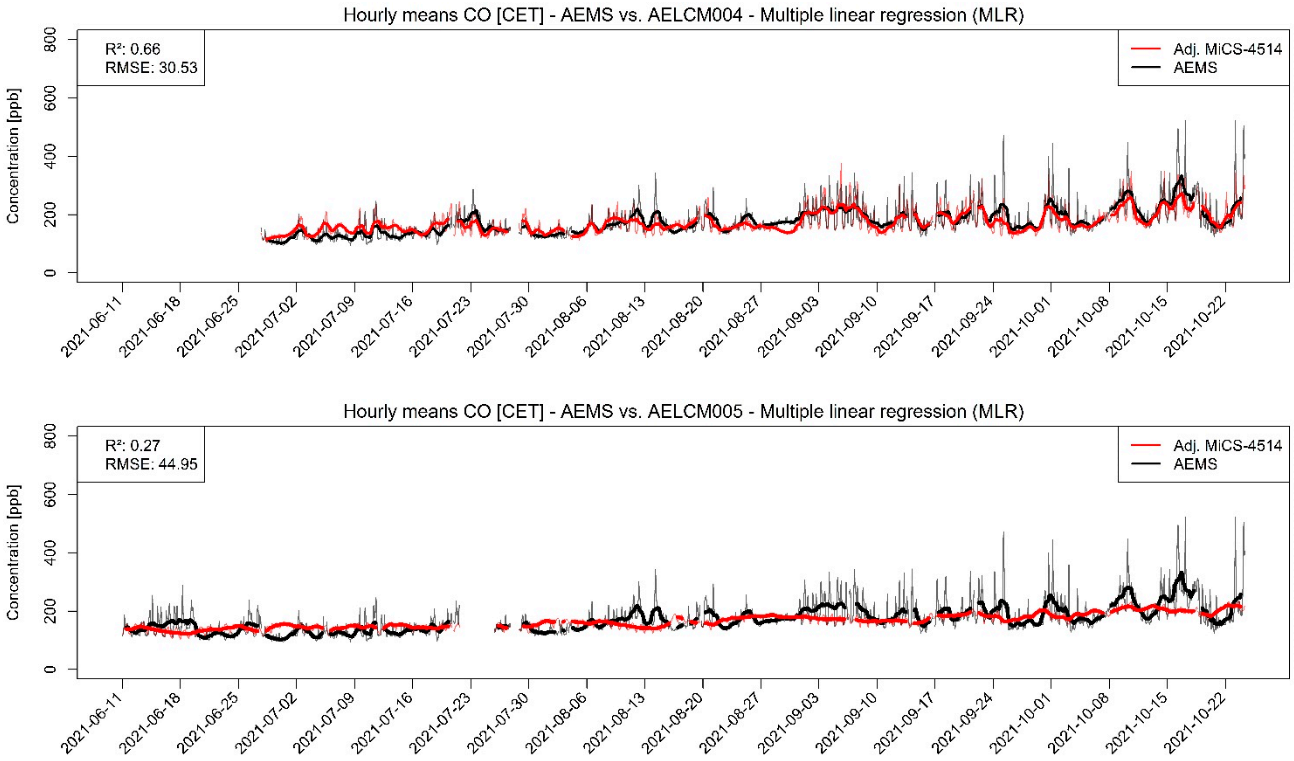

Figure S6.5. Model-adjusted hourly concentration means of different deployed MiCS-4514 using multiple linear regression vs. hourly concentration means of reference measurements given by the AEMS for ambient carbon monoxide in ppb. For smoothing the hourly concentration means a rolling 24-hour average is used: (**Top**) MiCS-4514 deployed in AELCM004; (**Bottom**) MiCS-4514 deployed in AELCM005.

S 7: Measured ambient ozone concentrations (AEMS vs. AELCM)

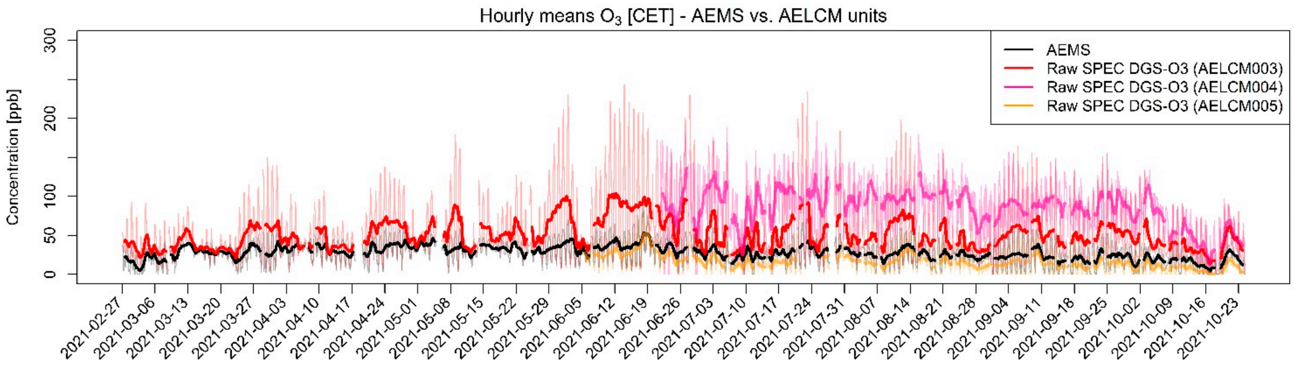

Figure S7.1. Raw hourly means of ambient ozone measured by SPEC DGS-O3 units deployed in AELCM003, AELCM004 and AELCM005 compared with hourly means of ambient ozone measurements given by the reference station AEMS. For smoothing the hourly concentration means a rolling 24-hour average is used.
